# The DEAD-box helicase Ded1 from yeast is an mRNP cap-associated protein that shuttles between the cytoplasm and nucleus

**DOI:** 10.1093/nar/gku584

**Published:** 2014-07-10

**Authors:** Meriem Senissar, Agnès Le Saux, Naïma Belgareh-Touzé, Céline Adam, Josette Banroques, N. Kyle Tanner

**Affiliations:** 1 Expression Génétique Microbienne, CNRS FRE3630 (UPR9073), in association with Université Paris Diderot, Sorbonne Paris Cité, Paris 75005, France; 2 Université Paris-Sud, Ecole Doctorale 426 GGC, Orsay, France; 3 Laboratoire de Biologie Moléculaire et Cellulaire des Eucaryotes, CNRS UMR8226 (FRE3354), UPMC, Paris 75005, France

## Abstract

The DEAD-box helicase Ded1 is an essential yeast protein that is closely related to mammalian DDX3 and to other DEAD-box proteins involved in developmental and cell cycle regulation. Ded1 is considered to be a translation-initiation factor that helps the 40S ribosome scan the mRNA from the 5′ 7-methylguanosine cap to the AUG start codon. We used IgG pull-down experiments, mass spectrometry analyses, genetic experiments, sucrose gradients, *in situ* localizations and enzymatic assays to show that Ded1 is a cap-associated protein that actively shuttles between the cytoplasm and the nucleus. NanoLC-MS/MS analyses of purified complexes show that Ded1 is present in both nuclear and cytoplasmic mRNPs. Ded1 physically interacts with purified components of the nuclear CBC and the cytoplasmic eIF4F complexes, and its enzymatic activity is stimulated by these factors. In addition, we show that Ded1 is genetically linked to these factors. Ded1 comigrates with these proteins on sucrose gradients, but treatment with rapamycin does not appreciably alter the distribution of Ded1; thus, most of the Ded1 is in stable mRNP complexes. We conclude that Ded1 is an mRNP cofactor of the cap complex that may function to remodel the different mRNPs and thereby regulate the expression of the mRNAs.

## INTRODUCTION

The DEAD-box proteins are an ubiquitous family of proteins that are found in nearly all organisms in all three kingdoms of life. They are implicated in all processes involving RNA, including transcription, splicing, ribosomal biogenesis, RNA export, translation and RNA decay [reviewed by (1–3)]. DEAD-box proteins belong to the DExD/H superfamily (SF2) of putative RNA and DNA helicases, and they are so named because of the amino acid sequence of motif II (D-E-A-D in single letter representation). The highly conserved ‘helicase’ core, which is found in all SF2 and SF1 proteins, consists of two, linked, RecA-like domains and sequence motifs that are conserved within the different families. Individual proteins are distinguished by the presence of nonconserved amino- and carboxyl-terminal extensions of highly variable length and by insertions and deletions within the conserved core (4).

The DEAD-box family contains at least nine conserved motifs that are needed for adenine recognition, and for ATP and RNA binding. The amino- and carboxyl-terminal extensions, as well as variability in the conserved motifs, enable researchers to classify the proteins into different subfamilies. Motif III links the cooperative binding of the ATP and RNA ligands, which subsequently promotes the ATPase activity of the protein in association with motifs II and VI (5). Thus, DEAD-box proteins are ATP-dependent RNA-binding proteins and RNA-dependent ATPases. In addition, a number of proteins studied *in vitro* were shown to unwind short RNA-RNA and RNA-DNA duplexes, but generally only at very high protein to substrate ratios. Typically, the proteins require a single-stranded region to load on the substrate prior to the displacement reaction, but this region can be either 5′ or 3′ to the duplex. Hence, most DEAD-box proteins lack directionality, and subsequently they are not processive.

These proteins are thought to have a number of cellular roles, such as helicases to disrupt RNA secondary structures, as chaperones to help form functional RNAs and as RNPases to facilitate the remodeling of ribonucleoprotein (RNP) complexes. However, the majority of the proteins that are tested *in vitro* lack substrate specificity and enzymatic regulation. This implies that the specificity and regulation are conferred by protein cofactors *in vivo*. Indeed, it is known that the intrinsically poor ATPase activity of Dbp5 (DDX19A), which is needed for messenger RNA (mRNA) export from the nucleus, is enhanced by Gle1 and phytic acid (inositol hexakisphosphate or IP6) on the cytoplasmic side of the nuclear pore [reviewed by (6–8)]. The inherently poor ATPase and helicase activity of eIF4A is significantly enhanced by the protein components of the translation-initiation complex [see (9) and references therein]. In contrast, the ATPase activity of the exon junction complex (EJC) protein eIF4AIII (DDX48) is inhibited by its interactions with MAGOH and Y14, while MLN51 enhances its affinity for RNA (10). Thus, the cofactors can function as on and off switches to control the enzymatic activities of the DEAD-box proteins. In contrast, little is known about how the substrate specificity is conferred. The exceptions are some eubacterial proteins that have accessory carboxyl-terminal domains with affinity for certain RNAs. For example, the *Escherichia coli* protein DbpA and the related YxiN protein from *Bacillus subtilis* have secondary binding sites for helix 92 of ribosomal RNA (rRNA) that are independent and separable from the helicase core domain (11). An opposing example is the SrmB protein from *E. coli* that has specificity for the 5′ end of 23S rRNA that is independent of its carboxyl-terminal domain (12). In this case, the specificity is at least partially conferred by its interactions with the L4 and L24 ribosomal proteins. Eukaryotic DEAD-box proteins often contain carboxyl-terminal extensions that enhance the affinity, in general, for RNAs, but to date these extensions are not known to confer specificity to discrete RNA substrates [(4,13) and references therein].

The budding yeast, *Saccharomyces cerevisiae*, has 25 DEAD-box proteins that are largely essential for the viability of the organism and that are not interchangeable [reviewed by (14)]. Many of the proteins are implicated in the biogenesis of the ribosomes, but the other proteins are involved in transcription, mRNA processing, RNA export, translation and RNA decay. Thus, yeast is a practical model system because the proteins are not redundant, they are mostly essential, they are represented in all the implicated cellular processes and there are obvious homologs in metazoans. Moreover, yeast genetics facilitate deletions of the endogenous gene and its replacement by a plasmid-encoded variant. Our laboratory has concentrated on studying Ded1, which has one of the highest DEAD-box protein activities (4).

Ded1 was first isolated as an essential domain in the chromosomal region encoding the *HIS3* gene [*DED1*: Defines Essential Domain1; (15)]. It was later found as an extragenic suppressor of a *prp8* mutation (16); Prp8 is a component of the U4/U6-U5 snRNP complex that is involved in mRNA splicing. Subsequently, Ded1 was implicated in the transcription of polymerase III RNAs (17), as a general translation-initiation factor (18,19), in 40S ribosome scanning (20), in yeast L-A virus replication (21) and finally in processing body (P-body) formation and RNA degradation (22). Ded1 is closely related to a subfamily of DEAD-box proteins that are involved in developmental and cell cycle regulation [reviewed by (23–26)], including the Drosophila Vasa protein for which the crystal structure was solved in the presence of RNA and a nonhydrolyzable analog of ATP [adenosine 5′-(β,γ-imido)triphosphate (AMP-PNP); (27)].

We used various genetic, physical and enzymatic approaches to isolate and characterize potential partners of Ded1, with the objective of better understanding the role(s) Ded1 plays in the cell, how its enzymatic activity is regulated and how the substrate specificity is determined. We found that Ded1 is physically and genetically associated with protein factors that are found on the 5′ cap structures of both nuclear and cytoplasmic RNP complexes, and that Ded1 actively shuttles between the two cellular compartments. We found that these factors stimulate the RNA-dependent ATPase activity of Ded1, which indicated that the interactions are functionally important. Finally, we found that Ded1 is associated primarily with stable RNPs that are not actively engaged in translation. This latter observation suggests that Ded1 might function to regulate RNP remodeling and thereby regulate genetic expression.

## MATERIALS AND METHODS

### Cloning, strains and molecular manipulations

Manipulations of yeast, including media preparation, growth conditions, transformation and 5-fluoroorotic acid (5-FOA) selection, were done according to standard procedures (28). Molecular manipulations, polymerase chain reaction (PCR) amplification and cloning were as previously described (29). We typically used the BY4742 yeast strain (Euroscarf) for the biochemical analyses and the CW04/W303 strains for the genetic studies (30). The *ded1::HIS3* strain was as previously described (31). It was transformed with plasmids expressing HA-Ded1 (29). Deletions and mutations of Ded1 and subcloning of the different genes were done by oligonucleotide-directed PCR. The *cbp20::KANMX4* strain (Euroscarf) was derived from BY4742. The *CBP20* gene in the pRSETC20 plasmid (32) was PCR amplified with oligonucleotides containing the SpeI and XhoI sites, cloned into the equivalent sites in the p415-PL and p424-PL plasmids (29) and transformed into the deletion strain. The expressed protein contained an amino-terminal HA tag. SpeI and XhoI sites were introduced into the *CBP80* pMalE clone (33) and then *CBP80* was cloned into the equivalent sites of the p415-PL plasmid. The *PL10* gene in the pRS315 plasmid with a *GDH* promoter and *PGK* terminator was as previously described (18). The *DDX3X* pTrcHis clone (34) was subcloned into SpeI-XhoI sites of p424-PL. The *BELLE* YEp181 construct was as previously described (35).

The GFP versions of *DED1* and *Δ30N-ded1*, containing SalI-XhoI sites, were subcloned into the SalI-cut pYM27 plasmid (Euroscarf Toolbox) containing the *EGFP* (enhanced green fluorescent protein) gene. This yielded proteins with carboxyl-terminal EGFP tags. SpeI-XhoI sites were introduced and the fusion construct was subcloned into the equivalent sites of p416-PL and p414-PL plasmids lacking the HA tags. The *Δ30N-ded1* also was cloned into the SphI-NheI sites of p416 containing the mCherry gene from pSL1 (36). The *EGFP* tag was integrated at the 3′ terminus of the *DED1* gene in the chromosome along with a *KANMX4* marker using pYM27 as previously described (37). The carboxyl-terminal GFP tag of Cbp80 was constructed by inserting SalI and XhoI sites into the pYM27 plasmid containing *CBP80* and then cloning the fragment into the XhoI-cut p414-PL and p416-PL plasmids. All constructs were verified by sequencing.

### *In situ* localization

Most of the *in situ* studies were done with the *mex67-5/xpo1-1* strain KWY610, where the expressed *mex67* and *xpo1* genes contained temperature-sensitive mutations [*mex67::HIS3(pUN100(CEN LEU2)mex67-5) xpo1::TRP1 xpo1-1::HIS3*; (38)]. Cells were incubated for 30–60 min at 36°C to block nuclear export. The *mex67-5* strain was treated similarly (39). The *crm1T539C* (*xpo1-T539C*) strain (40) was incubated for 1 h with 200 nM leptomycin. The wildtype G50 strain was an *ADE2* derivative of W303-1A (*ura3-1 trp1-1 leu2-2,112 his3-11,15 can1-100 RAD5 ADE2*; a gift from Michael Lisby). Cells were typically grown to an OD_595_ of ∼0.4 at 24–25°C.

Cells were fixed with 3.7% formaldehyde for 10 min with mixing, incubated for 10 min with 0.1 μg/ml DAPI (4′,6-diamidino-2-phenylindole), washed three times with deionized H_2_O and resuspended in H_2_O. Living cells were DAPI stained with 1 μg/ml, final, DAPI added to medium and incubated for 2–3 h with mixing. Cells were washed three times and resuspended in deionized H_2_O. Cells were visualized with a Carl Zeiss Axio Observer.Z1 microscope connected to an ORCA-R2 charge-coupled-device camera (Hamamatsu) using the Carl Zeiss AxioVision version 4.8.2 software. Images were edited using Adobe Photoshop CS3.

### Recombinant protein expression and purification

Ded1-His_6_ and eIF4A-His_6_ were expressed in pET22b (Novagen) and purified as previously described (5,29). Restriction sites were introduced by PCR into the pRSETC20 plasmid containing *CBP20* and the product was subcloned into the NdeI-BamHI sites of pET19b (Novagen). The His_6_-Cbp20 protein was purified as for the others. *GST-NAB2-HIS_6_* clone was described previously (41); the expressed protein was purified as for Ded1-His_6_ on nickel-agarose columns. *CDC33* (eIF4E) was cloned into the NdeI-BamHI sites of pET15b (Novagen) and *PAB1* in the XhoI-BamHI sites of pET19b; the expressed proteins contained amino-terminal His_6_ tags and they were purified as described for Ded1-His_6_. *GLE1* in the BG1805 plasmid (Open Biosystem) was subcloned into the BamHI site of pET19b. The sec63 domain of *BRR2* was cloned and expressed as previously described (42). The MalE-Cbp80 protein was expressed and purified as described elsewhere (33). The amino-terminal GST-tagged eIF4G1 fragments (1-596, 542-952, 882-952) were constructed by cloning the PCR-generated gene fragments into the BamHI-SmaI sites of pGEX-2T (GE Healthcare) and the expressed proteins were purified on glutathione Sepharose columns eluted with 10 mM reduced glutathione, 20 mM Tris–HCl, pH 7.5, 100 mM NaCl and 2 mM dithiothreitol (DTT). The gene coding for eIF4G9 in pGEX-1λT was expressed and the protein purified as previously described (43). All constructs were verified by sequencing.

### Immunoglobulin G-protein A Sepharose bead pull-down experiments

Cell extracts were made by harvesting cells at an OD_595_ around 0.6–0.8 by centrifugation, and resuspending the cells in a 40% volume (v/g) of a TpA buffer containing 10 mM 4-(2-Hydroxyethyl)piperazine-1-ethanesulfonic acid (HEPES), pH 7.9, 100 mM KCl, 0.5 mM DTT, 1.5 mM MgCl_2_ and 1X protease inhibitor cocktail (Roche Complete EDTA-free). Cells were then quick frozen by dripping the suspension in liquid nitrogen and the resulting cell beads were stored at −80°C until needed. The cells were ruptured while frozen with a Retsch MM 400 ball mill five times at 10 Hz for 3 min with a 25-mm steel ball. The grinding jar was cooled in liquid nitrogen between grindings. The cell powder was stored at −80°C until needed. The cells were thawed on ice and slowly made 200 mM in KCl over 30 min, with a 2 M stock solution, to lyse the nuclei. The extract was clarified by centrifugation for 30 min at 15 000 rpm in a Beckman JA20 rotor at 4°C. The supernatant was further clarified by centrifugation for 1 h at 37 000 rpm in a Beckman 60Ti rotor at 4°C. The supernatant was recovered, made 100 mM in KCl, made 10% in glycerol and frozen at −80°C until needed.

Ded1-immunoglobulin G (IgG) was crosslinked to protein A-Sepharose CL-4B beads (GE Healthcare) with glutaraldehyde as described elsewhere (44). We typically used 1–2 ml of cell extracts incubated with 30–75 μl of Ded1-IgG Sepharose beads. Generally, 1 μg/ml of RNAse A was added during the incubation. Material was generally incubated 1–2 h with mixing at 4°C. Beads were washed three times with 1 ml phosphate buffered saline (Euromedex), the bound proteins were eluted with 400 μl of 200 mM glycine, pH 2.5, for 15–30 min with mixing at room temperature. The solution was titrated to a neutral pH with concentrated NaOH, made 150 μg/ml in sodium deoxycholate and the proteins were precipitated using 15%, final, trichloroacetic acid (TCA). The pellet was washed with cold acetone, dried and resuspended in loading buffer containing 67 mM Tris–HCl, pH 6.8, 1.3% sodium dodecyl sulphate (SDS), 2% β-mercaptoethanol, 0.07% bromophenol blue and 10% glycerol. The eluted proteins were separated on SDS-Laemmli gels, transferred to Hybond-C super membranes (Amersham Biosciences) and probed with IgG against Ded1 (31), eIF4A (a gift from P. Linder), eIF4E (45), HA tag (Covance), Cbp80 (46), Dhh1 (47) Nab2 (Santa Cruz Biotechnology), eIF4G1 and eIF4G2 (48), Pab1 (49) and Gle1 (50). Horseradish peroxidase-conjugated antimouse (Promega), antirabbit (Promega) and antigoat (Santa Cruz Biotechnology) were used as secondary antibodies, and the signals were revealed with a Clarity Western ECL Substrate kit (Bio-Rad).

Proteins were identified by nano-liquid-chromotagraphy-electronspray mass spectrometry (nanoLC-MS/MS) using the Plateforme Protéomique de Strasbourg service at the University of Strasbourg. For the pull-down experiments of yeast extracts, the proteins were electrophoretically separated on a 10% SDS-Laemmli polyacrylamide gel, the gel was stained with colloidal Brilliant Blue G (Sigma) and the entire gel was sent to the service. The service cut each lane of the gel into 48 pieces and then incubated the pieces with trypsin. The eluants were combined into six fractions that were injected into the nanoLC-MS/MS.

### Other pull-down experiments

We used 7 μl of 7-methyl-GTP Sepharose beads (GE Healthcare) equilibrated in 100 μl of TpB buffer containing 20 mM HEPES, pH 7.9, 100 mM KCl, 2 mM MgCl_2_ and 2 mM DTT. The beads were then incubated with 5 μg of purified eIF4E or Cbp20 for 90 min on ice and then washed three times with 1 ml of TpB buffer. We added 5 μg of the purified proteins of interest with 50 U of RNase I, incubated 90 min on ice, washed three times with 1 ml TpB buffer and eluted the bound proteins with 15 μl of 1 mM 7-methyl-GTP (Sigma) over 90 min at 4°C. The same protocol was used for the pull-down experiments with glutathione Sepharose 4B (GE Healthcare) and amylose resin (BioLabs) except the bound proteins were eluted with 15 μl of 10 mM reduced glutathione and 15 μl of 10 mM maltose, respectively.

### *In vitro* ATPase assays

The ATPase reactions were as previously described except that the currently available whole yeast RNA from Sigma (type III) contained inhibitors (4). Consequently, we use whole yeast RNA from Roche, which was phenol extracted and fractionated on a DEAE-Sephadex column using a NaCl gradient to remove inhibitors that were present. The fractions that stimulated the ATPase activity the most were combined and used for the subsequent assays. We typically used 0.45 μg/μl in the reaction. Assays were done with a defined RNA substrate using a T7-RNA-polymerase runoff transcript of an EcoRI-cut plasmid containing the 3′ untranslated region (UTR) of the *CYC1* gene as previously described (51). Transcription was done with the T7-MEGAshortscript kit (Ambion). This 202 nucleotide-long RNA was capped and polyadenylated with the T7 mScript Standard mRNA kit (Cellscript). Proteins were preincubated in the reaction mix containing the RNA for 20 min on ice. Samples were then placed at 30°C, and the reactions were initiated by making them 1 mM in ATP.

### Polysome profiles

Yeast cultures were grown to an OD_595_ of ∼0.8 at 30°C and then cycloheximide (Sigma) was added to a final concentration of 100 μg/ml. The cultures were incubated with mixing on ice for 10–15 min, and the cells were harvested by centrifugation. In the rapamycin experiments, the cultures were made 0.5 μg/ml in rapamycin (InvivoGen) at an OD_595_ of ∼0.5 and then incubated ∼2 h at 30°C prior to adding the cycloheximide. Cells from 500-ml cultures were collected by centrifugation, washed and the pellets resuspended in 500 μl of a lysis buffer containing 20 mM Tris–HCl, pH 7.5, 50 mM NaCl, 5 mM MgCl_2_, 1 mM DTT and 0.1 mM ethylenediaminetetraacetic acid. Cells were frozen at −20°C until needed.

Cells were extracted with a FastPrep-24 homogenizer (MP Biomedical) in the presence of 1X protease inhibitor cocktail using a dry ice cooled rotor and acid-washed glass beads (Sigma; 425–600 μm). Samples were lysed at 6.5 M/S four times for 30 s, with the rotor cooled between extractions. Extracts were passed through MiniBioSpin columns (BioRad) and then clarified by centrifugation two times for 10 min at 12 000 g in an Eppendorf 5415R centrifuge. The resulting supernatants were loaded onto 12 ml gradients, consisting of 7–47% sucrose in lysis buffer, at an OD_260_ of 10–12 (100–200 μl of extract) and then centrifuged in an SW41ti rotor (Beckman) at 39 000 rpm for 3 h at 4°C. Half milliliter fractions were collected from the top of the gradients with a Retriever 500 (ISCO) fraction collector and a Type 11 Optical unit (ISCO) with 254-nm filters. Generally, the proteins were precipitated by making the solutions 150 μg/ml, final, in sodium deoxycholate, then adding TCA to 15% final. The solutions were centrifuged, and the pellets washed with cold acetone, dried and resuspended in loading buffer. The recovered proteins were electrophoretically separated by SDS-Laemmli gels, transferred to membranes and then identified with various IgGs (see above). Samples analyzed by nanoLC-MS/MS were first further purified by Ded1-IgG pull downs, washed and precipitated as described above. The resulting dried pellets were then sent to the Plateforme Protéomique de Strasbourg service for analysis.

## RESULTS

### Functional homologs of Ded1

Because Ded1 is a homolog of a subset of DEAD-box proteins involved in cell cycle and developmental regulation in other organisms, we were interested in determining the degree of functional complementation of this subfamily of proteins in yeast, which might help clarify the role(s) of Ded1 *in vivo*. Drosophila Belle (35), mouse PL10 (18), *Schizosaccharomyces pombe* Ded1 (52) and human DDX3X (53) were shown to complement yeast cells deleted for Ded1. Likewise, we previously showed that the more distantly related Vasa protein from Drosophila could weakly complement yeast (5). However, the degree of complementation was not evident for DDX3 and PL10. In these experiments, we directly compared the complementation of PL10, DDX3X and Belle with Ded1.

When selected on plates containing 5-FOA, all three proteins could complement the deletion of Ded1, but with significant differences (Supplemental Figure S1). PL10 showed the best growth, DDX3X supported weaker growth, while Belle poorly complemented the Ded1 deletion. Cells transformed with the Vasa gene showed no apparent growth after 3 days, but slight growth was apparent after longer incubation times (5). The Belle construct was expressed with the Ded1 promoter and terminator, while the other constructs were expressed with strong constitutive promoters. We previously showed that Ded1 and other yeast DEAD-box proteins inhibit growth when highly expressed (4). This is consistent with the reduced complementation of Ded1 when expressed off the high-copy plasmid with the strong alcohol dehydrogenase (ADH) promoter relative to the equivalent construct in the low-copy plasmid (Supplemental Figure S1). However, no difference was seen when Belle or DDX3 was expressed off low- and high-copy plasmids. Hence, complementation by the Ded1 homologs was probably not significantly affected by the level of protein expression. Nevertheless, we cannot rule out a combination of both stimulatory and inhibitory effects of the homologs in these assays. Overexpression of the unrelated DEAD-box proteins eIF4A, which also is implicated in translation initiation, and Dhh1 failed to support growth (Supplemental Figure S1). Thus, only the tested Ded1 homologs could partially replace Ded1 in the cell, which pointed to a functional conservation of the proteins among eukaryotes.

### Ded1 shuttled between the cytoplasm and the nucleus

The Ded1 subfamily of proteins contains a leucine-rich nuclear export signal (NES) on the amino terminus (54). Both Xenopus An3 and human DDX3X are known to shuttle between the cytoplasm and nucleus (54–58), but Ded1 was previously shown to be predominately located in the cytoplasm (18). Nevertheless, it was possible that Ded1 shuttled between the nucleus and cytoplasm at low levels that were not readily detectable by the indirect immunofluorescence that was used in those experiments. To test this, we constructed a fusion protein between Ded1 and the GFP that was expressed from the low-copy p416-PL plasmid. This construct supported nearly wildtype growth at 18 and 30°C in cells deleted for wildtype *DED1*, but it showed slightly reduced growth at 36°C (Supplemental Figure S2). Similar results were obtained with the plasmid-expressed HA-tagged Ded1, which suggested that the GFP tag, *per se*, did not significantly alter the properties of Ded1. To verify this, we integrated the *GFP* gene at the 3′ terminus of *DED1* in the chromosome. In this case, cells grew like the wildtype strain at all the temperatures tested (Supplemental Figure S2). Thus, the higher, or constitutive, expression levels of the plasmid-encoded proteins probably caused the slightly reduced growth at 36°C.

**Figure 1.**
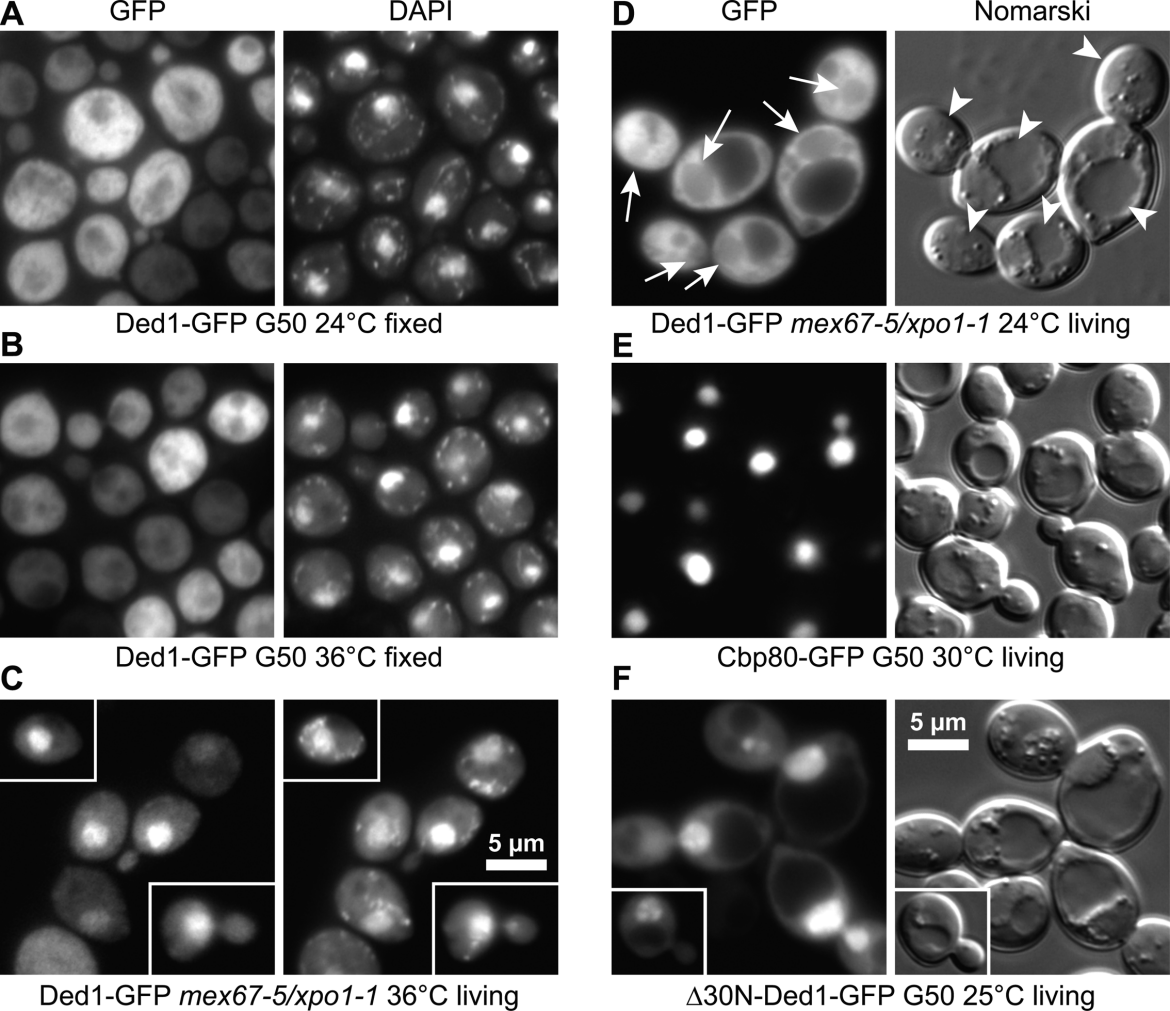
Ded1 shuttled between the cytoplasm and nucleus. (**A**) and (**B**) Fluorescence microscopy of cells that were fixed with formaldehyde when stained with DAPI to reveal the nuclei and mitochondria (seen as speckles). Ded1-GFP was primarily located in the cytoplasm of wildtype (G50) cells at all the temperatures tested. (**C**) In contrast, Ded1-GFP shows a strong nuclear location in the *mex67-5/xpo1-1* mutant when incubated at nonpermissive temperatures for 30 min in living cells stained with DAPI. (**D**) Fluorescence microscopy (GFP) and Nomarski interference contrast microscopy of living yeast cells. Ded1-GFP was mostly located in the cytoplasm of *mex67-5/xpo1-1* cells at the permissive temperatures (24°C), but a small amount was visible in the nuclei as well (arrows). The vacuoles/lysosomes are indicated with arrowheads. (**E**) In contrast, Cbp80-GFP was almost exclusively located in the nuclei. (**F**) Ded1-GFP that was deleted for the 30 amino-terminal residues containing the NES sequence (Δ30N) and the eIF4E binding site accumulated in the nuclei of yeast even in wildtype cells.

We next asked where the Ded1-GFP fusion protein was located within the cell. Fluorescence microscopy of living cells revealed that the protein was primarily located within the cytoplasm, but the nuclei showed a faint signal relative to the vacuoles/lysosomes. To verify this, we fixed the cells and then stained them with DAPI to identify the nuclei (Figure 1A and B). These experiments confirmed that Ded1 was largely excluded from the nuclei, but that the faint signals corresponded to the nuclei rather than to the vacuoles. Both plasmid-expressed and integrated Ded1-GFP showed the same distributions, which indicated that the expression levels did not alter the results. However, the intensity of the Ded1-GFP signal varied between cells in both the plasmid-borne and integrated cells. This implied that the expression levels of Ded1 were variable between cells (Figure 1A and B and Supplemental Figure S3). Thus, our results were consistent with the possibility that a small amount of Ded1 was located in the nucleus.

Proteins shuttle between the nucleus and cytoplasm through the nuclear export receptor Crm1, also known as Xpo1 in yeast, which recognizes the NES of the proteins and that is part of the nuclear pore complex (59). We reasoned that Ded1 may be maintained at low concentrations in the nucleus, and that this regulation may be mediated through Xpo1. Crm1 is selectively inhibited by the antibiotic leptomycin B from *Streptomyces* (60), but Xpo1 from *S. cerevisiae* is resistant to this drug. Therefore, we used a mutant strain where the *XPO1* gene was deleted and the plasmid expressed copy contained a threonine to cysteine mutation at position 539 that conferred sensitivity to the drug (40). We transformed this strain with the *DED1-GFP* plasmid and analyzed the distribution of the protein after treatment of the cells with 200 nM of leptomycin B by fluorescence microscopy (Supplemental Figure S3). The cells showed a high degree of variability, but a few percent of the cells showed a very strong accumulation of Ded1-GFP in the nuclei. This indicated that Ded1 did shuttle between the nucleus and cytoplasm, but that Ded1 did not use exclusively the Xpo1/Crm1 pathway.

The mRNAs are typically exported from the nucleus through another transport system involving Mex67 [TAP (Tip-associated protein) in metazoans; reviewed by (61)]. It was possible that Ded1 associated with mRNAs also was being transported through this pathway. Therefore, we used a different yeast strain that had both *XPO1* and *MEX67* deleted from the chromosome and that expressed temperature-sensitive variants of the proteins from plasmids [*mex67-5/xpo1-1*; (38)]. When we transformed this strain with the *DED1-GFP* plasmid, we saw very intense fluorescence in the nuclei at the nonpermissive temperatures in the majority of cells (>50%, Figure 1C), but not at the permissive temperatures (Figure 1D). Moreover, the accumulation of Ded1 in the nuclei was reversible; cells incubated at 36°C and then left at room temperature for 30 min showed little Ded1-GFP in the nuclei. This meant that the transport of Ded1 was highly dynamic. Both the Xpo1 and Mex67 pathways were used because a strain expressing only the *mex67-5* mutant showed only a few percent of the cells with Ded1-GFP strongly accumulated in the nuclei (Supplemental Figure S3). Thus, there were clearly cell-to-cell differences that suggested that the primary export pathway varied between cells.

Transport was not strongly dependent on the enzymatic activity of Ded1 because a Ded1-GFP variant containing the P-loop mutation K192A showed only a small increase in some of the nuclei of the *mex67-5/xpo1-1* strain under permissive conditions (data not shown). This mutation eliminates the ATPase activity and reduces the RNA-binding affinity of Ded1 *in vitro* (4). This result was in contrast to that previously obtained for An3, where Crm1-dependent export was significantly reduced with a protein mutated in motif II (62). However, we cannot rule out the possibilities that some cells were more dependent on the enzymatic activity of Ded1 for nuclear export than others or that only one pathway was dependent on this activity. In contrast, no differences were seen between the wildtype and the K192A mutant Ded1-GFP at the nonpermissive temperatures, which indicated that the nuclear import of the Ded1-GFP was not strongly affected (data not shown).

As a control, we looked at the cellular location of the GFP-tagged, cap binding protein Cbp80, which is known to be predominately a nuclear protein (63). Consistent with this, Cbp80-GFP was almost exclusively found in the nuclei (Figure 1E). This was in contrast to Ded1-GFP where a detectable signal was always present in both the nucleus and cytoplasm under all conditions (Figure 1A–D). Finally, we obtained very strong nuclear localization, even in the wildtype cells, when we deleted the 30 amino-terminal residues containing both the eIF4E-binding motif and the NES sequence from Ded1 (Figure 1F). However, there was significant cell-to-cell variability that ranged from little accumulation to very high. The nuclear-accumulated Ded1 colocalized with Cbp80 (Supplemental Figure S4). These data demonstrated that Ded1 shuttles between the nucleus and cytoplasm, and that it is exported by both the Mex67- and Xpo1-dependent nuclear-export complexes in cells growing at early exponential phase. Similar results were obtained with DDX3X (55,56,58). Thus, it was likely that Ded1 formed complexes with both nuclear and cytoplasmic factors, and that the Ded1 exported through the Mex67-dependent pathway was probably associated with mRNAs as part of an messenger ribonucleoprotein (mRNP) complex. Likewise, a subset of mRNPs are transported through the Xpo1/Crm1 pathway [reviewed by (64)], although this is a relatively minor pathway in yeast (40). DDX3X was found to be exported by this pathway in mRNPs bound with eIF4E instead of the cap binding complex (CBC) proteins (58). In contrast, because Ded1 contains a NES, it may be maintained at low levels in the nucleus through a karyopherin-mediated export by the Xpo1-dependent pathway. Finally, it also was possible that Ded1 was associated with the export of rRNAs, which involves both the Mex67 and Xpo1 proteins (64).

### Ded1 genetically interacted with cytoplasmic and nuclear mRNP factors

DDX3X interacts with some components of the cytoplasmic, cap binding, eIF4F complex, which is involved in translation initiation of mRNAs (26,65). The eIF4F complex consists of the 7-methylguanosine binding protein eIF4E, the scaffolding protein eIF4G, and the DEAD-box helicase eIF4A [reviewed by (9)]. The eIF4G protein exists as two isoforms in yeast consisting of a highly-expressed eIF4G1 and a lesser-expressed eIF4G2. The polyadenosine binding protein Pab1 binds eIF4G and thereby loops back the mRNA to form a circle. It was shown recently that Ded1 physically interacts with the carboxyl-terminal domain of eIF4G1 (66). The nucleus contains an analogous structure called the nuclear cap binding complex [CBC; reviewed by (9,67)]. This complex consists of the 7-methylguanosine binding protein Cbp20 and an accessory protein, Cbp80. DDX3X in the nucleus was found associated with the nuclear CBC proteins (26,56,58,68,69). In addition, the nucleus contains the polyadenosine binding protein Nab2 that is involved in regulating polyadenylation of mRNAs and in mRNA export [reviewed by (70)].

**Figure 2.**
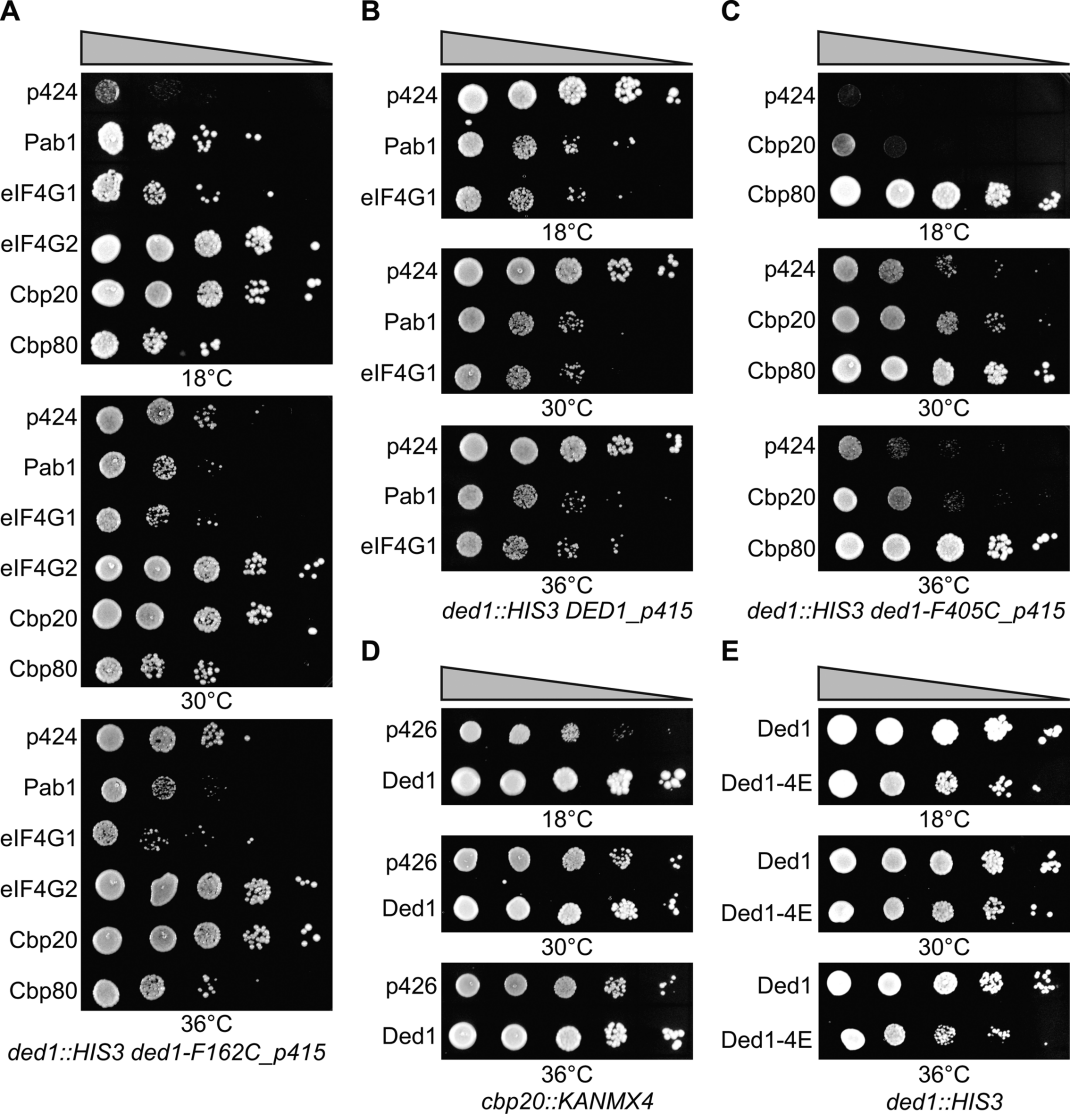
Cap-associated factors were genetically linked to Ded1. Cells transformed with plasmids expressing the indicated proteins were serially diluted by factors of 10, spotted on synthetic minimal-medium plates lacking tryptophan (p424 plasmids) or uracil (p426 plasmids) and incubated at the indicated temperatures. Plates were incubated for 3 days at 30 and 36°C, and for 8 days at 18°C. (**A**) Cells expressing the slow-growth mutant Ded1-F162C of the Q motif were transformed with plasmids overexpressing the indicated proteins. The p424 was an empty plasmid control. (**B**) Pab1 and eIF4G1 were inhibitory when overexpressed in cells expressing wildtype Ded1. (**C**) Multicopy suppression of cells expressing the slow-growth mutant Ded1-F405C of motif IV. Cells were transformed with plasmids overexpressing Cbp20 and Cbp80. (**D**) Ded1 was a multicopy suppressor of cells deleted for *cbp20*. (**E**) Cells expressing Ded1 mutated in the eIF4E-binding motif (Ded1-4E; Y21A/L26A) gave a slow-growth phenotype relative to the wild type (Ded1) when expressed off the low copy p415-PL plasmid.

We reasoned that, since Ded1 was present in the nucleus, it might interact with the nuclear cap-binding complex. To test for this, we used a slow-growth variant of Ded1 that was mutated in the Q motif involved in adenine binding of ATP [Ded1-F162C; (71)]. We then overexpressed various candidate proteins to see if they could partially restore the growth phenotype (Figure 2A). The overexpressed Cbp20 and eIF4G2 proteins restored the growth to nearly wildtype levels. Cbp80 partially restored growth, while Pab1 and eIF4G1 promoted growth at 18°C but inhibited growth at the higher temperatures. This latter result pointed to a synthetic lethal (inhibitory) effect of the overexpressed proteins. Indeed, we saw growth inhibition at all temperatures when we overexpressed Pab1 and eIF4G1 in a strain expressing the wildtype Ded1, which indicated that the inhibitory effects were unrelated to Ded1 (Figure 2B). Thus, all five of the tested proteins interacted genetically with Ded1.

Oddly, the multicopy suppression of Ded1 mutants by the other proteins was dependent on the location of the mutation. Thus, in contrast to the preceding results, we found that a slow-growth mutant in motif IV (F405C) was better suppressed with Cbp80 than with Cbp20 (Figure 2C). Motif IV mutants dissociate the cooperative binding between ATP and the RNA substrates (72). However, there was no strong correlation between the multicopy suppression and the specific motif mutated; a different Q motif mutant, T166Q, was better suppressed by Cbp80 than by Cbp20 (data not shown).

The multicopy suppression worked in reverse for some of the proteins; i.e. overexpressed Ded1 was able to suppress the slow-growth phenotype of a strain deleted for Cbp20 (Figure 2D). The strain deleted for Cbp80 showed a slight effect with overexpressed Ded1, but this deletion strain had a more severe phenotype than for Cbp20 (data not shown). Finally, we obtained a slight slow-growth phenotype when we mutated the putative eIF4E-binding site on Ded1 (Y21A/L26A; Figure 2E). This was consistent with a previously demonstrated genetic link to eIF4E (19). These genetic results were compatible with Ded1 interacting with both cytoplasmic and nuclear cap-binding complexes.

### Ded1 was associated with large RNP complexes

**Figure 3.**
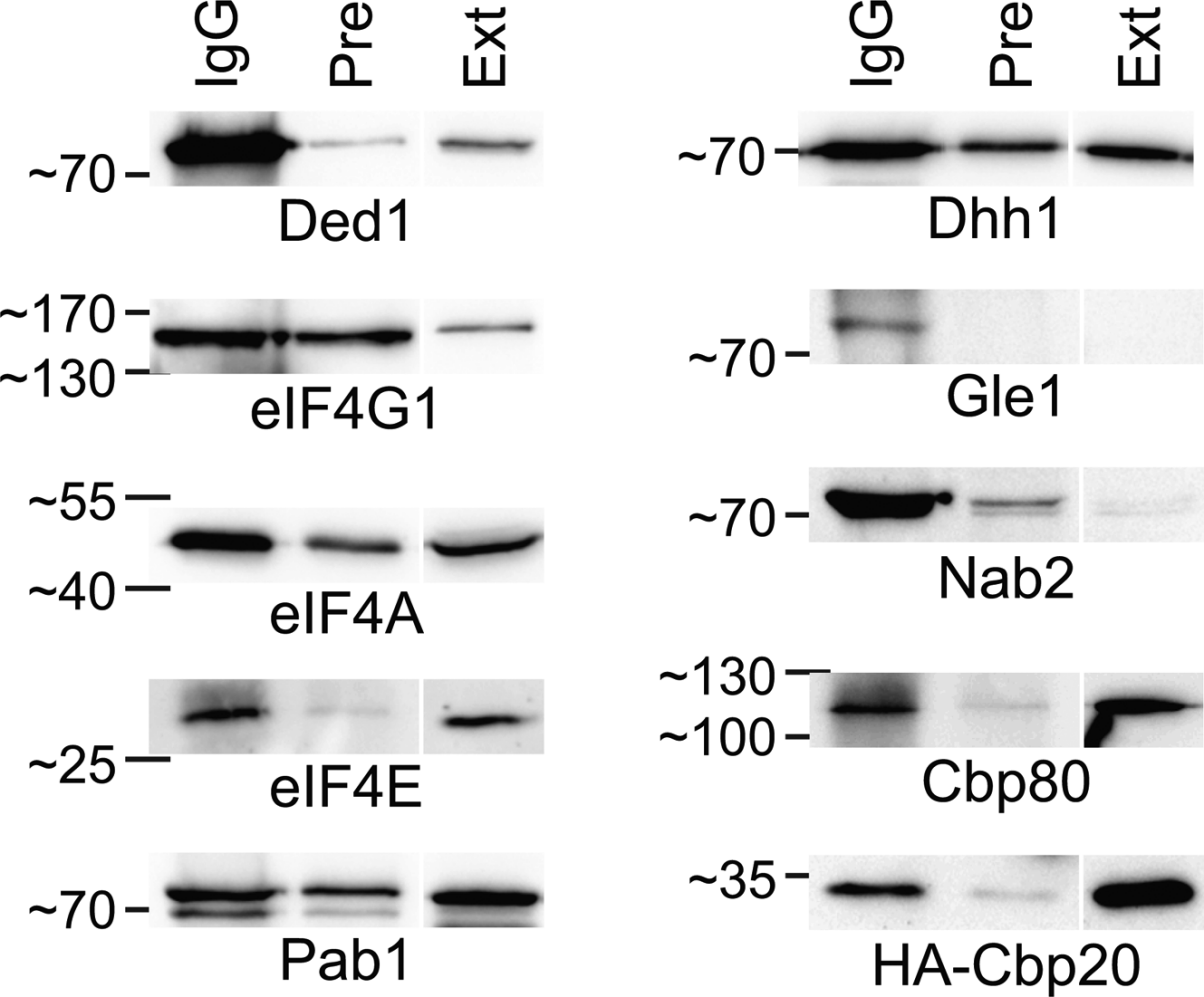
Ded1-IgG pull-down experiments. Yeast extracts were incubated with Ded1-IgG-Sepharose beads, washed, the proteins eluted and then the recovered proteins were separated by an 8% SDS polyacrylamide gel electrophoresis (PAGE) for Cbp80 and eIF4G1, and by an 10% SDS PAGE for the others. IgG, rabbit serum after induction with injected, purified, Ded1 protein; Pre, rabbit pre-immune serum; Ext, the equivalent of 1 μl of yeast extract. Note that for each set of experiments, the material was analyzed on the same gel with the same extract; an empty lane was placed between the pull-down lanes and the extract lanes to avoid potential cross-contamination. The separated proteins were transferred to membranes, cut into strips and probed with antibodies against the indicated proteins. Cbp20 was expressed from a plasmid incorporating an amino-terminal HA tag, and it was detected with IgG against HA. The other proteins were detected from the wildtype BY4742 strain with antibodies specific to each protein. The molecule weight markers were PAGE Ruler from Thermos Scientific.

We next asked what were the factors that were physically associated with Ded1 in the cell. We initially tried the double-affinity purification procedure that uses Ded1 fused to the protein A and the calmodulin-binding proteins to isolate complexes containing Ded1 from cell extracts [TAP-tag method; (73)]. However, the long loading times on the columns and poor recovery of Ded1 precluded reliable identification of the cofactors. Nevertheless, the results from the MALDI-TOF MS (matrix-assisted laser desorption/ionization time of flight mass spectrometry) analyses were consistent with Ded1 interacting with the translational machinery (data not shown). Therefore, we used IgG against Ded1, attached to protein A-Sepharose beads, to pull down the complexes in yeast extracts. We tried various conditions and found that light digestion of the extracts with RNase A gave the best yields and quality of material. Western blot analyses of the separated proteins revealed the expected proteins eIF4E, eIF4G1, Pab1, Cbp20, Cbp80 and Nab2 (Figure 3). We also recovered a weak signal for eIF4G2 in some experiments. Ded1 was previously found to interact with Dhh1 and Gle1 (74,75), and we were able to identify these proteins as well (Figure 3). Moreover, we recovered eIF4A. Thus, Ded1 was associated with both nuclear and cytoplasmic proteins that are known components of mRNA RNPs. These results were verified by nanoLC-MS/MS analysis of the polyacrylamide gels (Supplemental Tables S1 and S2).

There was significant variability in the degree of enrichment between the different proteins relative to the crude extract and the pre-immune IgG. Pab1 was only slightly enriched, while eIF4E was strongly enriched. The expression levels of the different proteins vary enormously, from nearly 200 000 copies per cell for Pab1 to about 1000 copies per cell for Gle1 (http://yeastgenome.org). Ded1 was calculated to be present at about 30 000 copies per cell (76). This partially accounted for the difference in enrichment on the IgG beads relative to the extract. However, the cytoplasmic eIF4F-associated proteins were clearly more enriched relative to the extract than the nuclear CBC complex proteins. This was consistent with Ded1 having primarily a cytoplasmic location, as was seen with the Ded1-GFP within the cell. The exception was Nab2 that was highly enriched, even though it is typically located primarily in the nucleus [Figure 3; (70)]. Nab2 is present at about 10 000 copies per cell (http://yeastgenome.org). Thus, the stoichiometry of the different proteins was complex, which indicated that multiple different RNPs were present. Moreover, the binding affinities of the different factors were likely different, which might result in the preferential loss or retention of certain factors relative to the others from the isolated complexes. Nevertheless, the data were consistent with Ded1 binding to both nuclear and cytoplasmic RNP complexes.

### Ded1 was a 7-methylguanosine cap-associated factor

**Figure 4.**
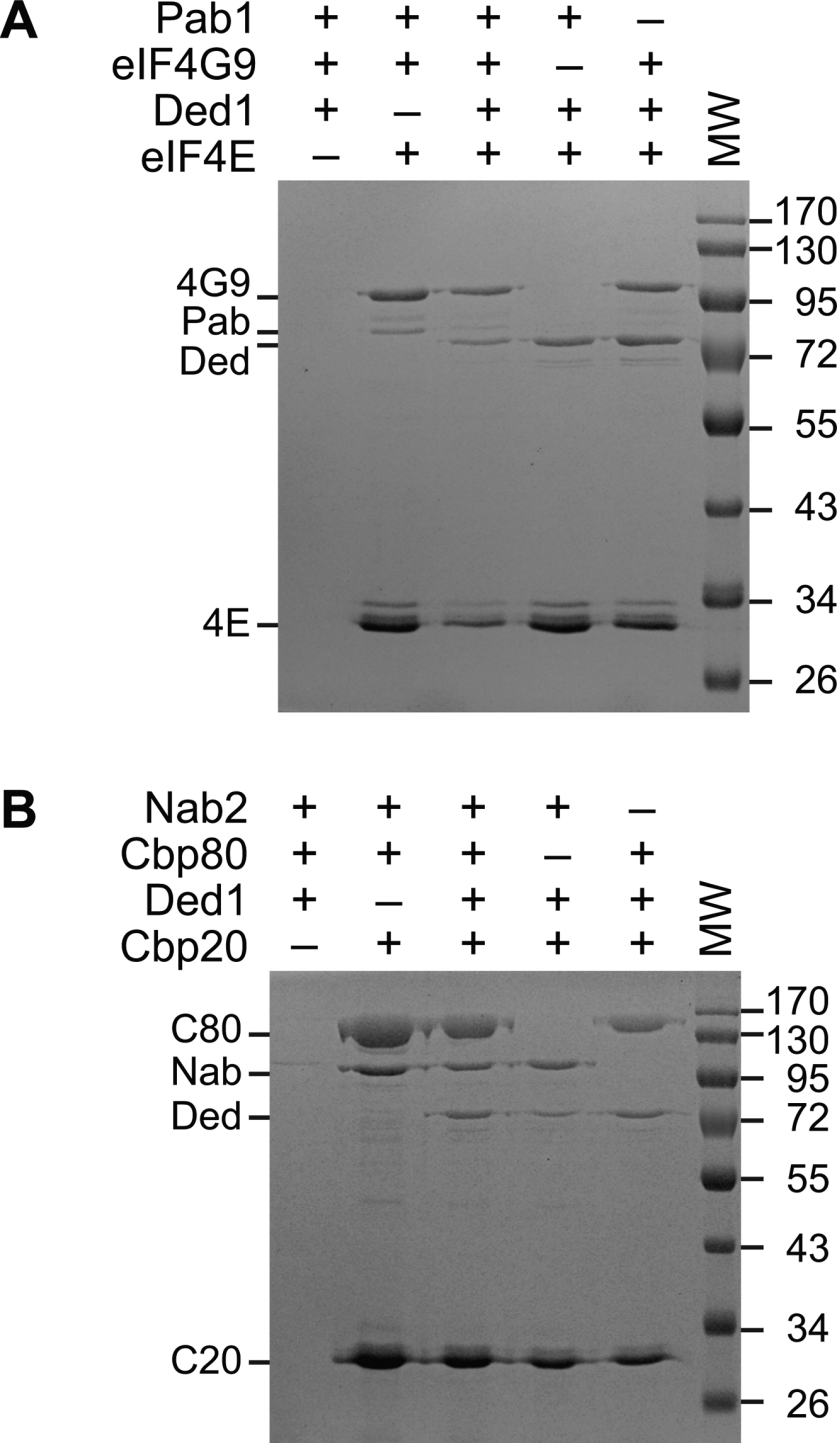
Ded1 was retained by cytoplasmic and nuclear cap-binding complexes. The indicated proteins were incubated with 7-methyl-GTP Sepharose beads, washed and then eluted with 1 mM free 7-methyl-GTP. The eluted proteins were separated by electrophoresis on 10% SDS polyacrylamide gels (SDS PAGE) and stained with Coomassie blue. (**A**) Proteins associated with the predominately cytoplasmic eIF4E protein bound to the beads. The eIF4G9 protein consisted of amino acids 160–492 of eIF4G1. Pab1 (Pab) gave a weak signal, but its presence was verified by western blot analysis. (**B**) Proteins associated with the predominately nuclear Cbp20 protein bound to the beads. Nab2 had a very weak affinity for the beads in the absence of the other proteins. The markers reflect the sizes of the Prestain Protein Ladder (Euromedex).

The preceding experiments indicated that Ded1 was in complexes containing the identified proteins, but they did not demonstrate a direct or functional interaction between the proteins. However, it seemed likely that Ded1 was part of the nuclear and cytoplasmic cap-binding complexes. To test this, we purified the different recombinant proteins from *E. coli* (Supplemental Figure S5) and tested their ability to form complexes on 7-methyl-GTP Sepharose beads. Both Cbp20 and eIF4E have high affinity for 7-methylguanosine. We bound these proteins to the beads, added various combinations of the purified proteins and then eluted the proteins retained (Figure 4). To facilitate the analyses, we used a shorten version of eIF4G1 (eIF4G9; amino acids 160–492) that was known to retain its capacity to interact with Pab1 and eIF4E (43). Of the proteins tested, only the polyadenosine-binding protein Nab2 showed a weak, nonspecific affinity for the beads.

As expected, eIF4G9 and Pab1 formed a complex with eIF4E, although the interactions with Pab1 appeared to be weaker (Figure 4A). Pab1 interacts indirectly with eIF4E through the scaffolding protein eIF4G, and it is normally bound to the polyadenosine tail of mRNAs, which significantly enhances its affinity for eIF4G (77). Ded1 was retained by eIF4E under all the protein combinations tested, which indicated that Ded1 directly contacted eIF4E. However, we reproducibly recovered less protein when Pab1 was present with Ded1 in the mixture. This suggested that Ded1 and Pab1 had overlapping binding sites with the other factor(s), which were most likely mediated by eIF4G1. Thus, we cannot rule out the possibility that the lower recovery of all four proteins reflected a heterogeneous mix of complexes constituting different combinations of the proteins.

We obtained similar results for proteins binding to Cbp20 (Figure 4B). As expected, Cbp80 showed a high affinity for Cbp20, but surprisingly we also obtained an interaction with Nab2. The interactions between Nab2 and Cbp20 are less well characterized than those between Pab1 and eIF4G, but they were thought to be mediated by the RNA because RNase-treated extracts showed reduced interactions between the proteins (78,79). Our results indicated that Nab2 was capable of forming direct protein–protein interactions as well. Thus, Nab2 may fold-back the mRNA in the nucleus in a way analogous to what Pab1 does in the cytoplasm. Regardless, Ded1 was weakly associated with all the combinations of proteins, which indicated that Ded1 formed direct protein-to-protein interactions with Cbp20. Both the eIF4E and the CBC complexes were resistant to both RNase A and RNase I, which showed that the interactions were not mediated by contaminating RNAs bound to the purified proteins. These results were consistent with the previous observation that Ded1 binds eIF4E-eIF4G1 in an RNA-independent fashion on 7-methyl-GTP Sepharose beads (66).

### Ded1 physically interacted with mRNP cap factors

**Figure 5.**
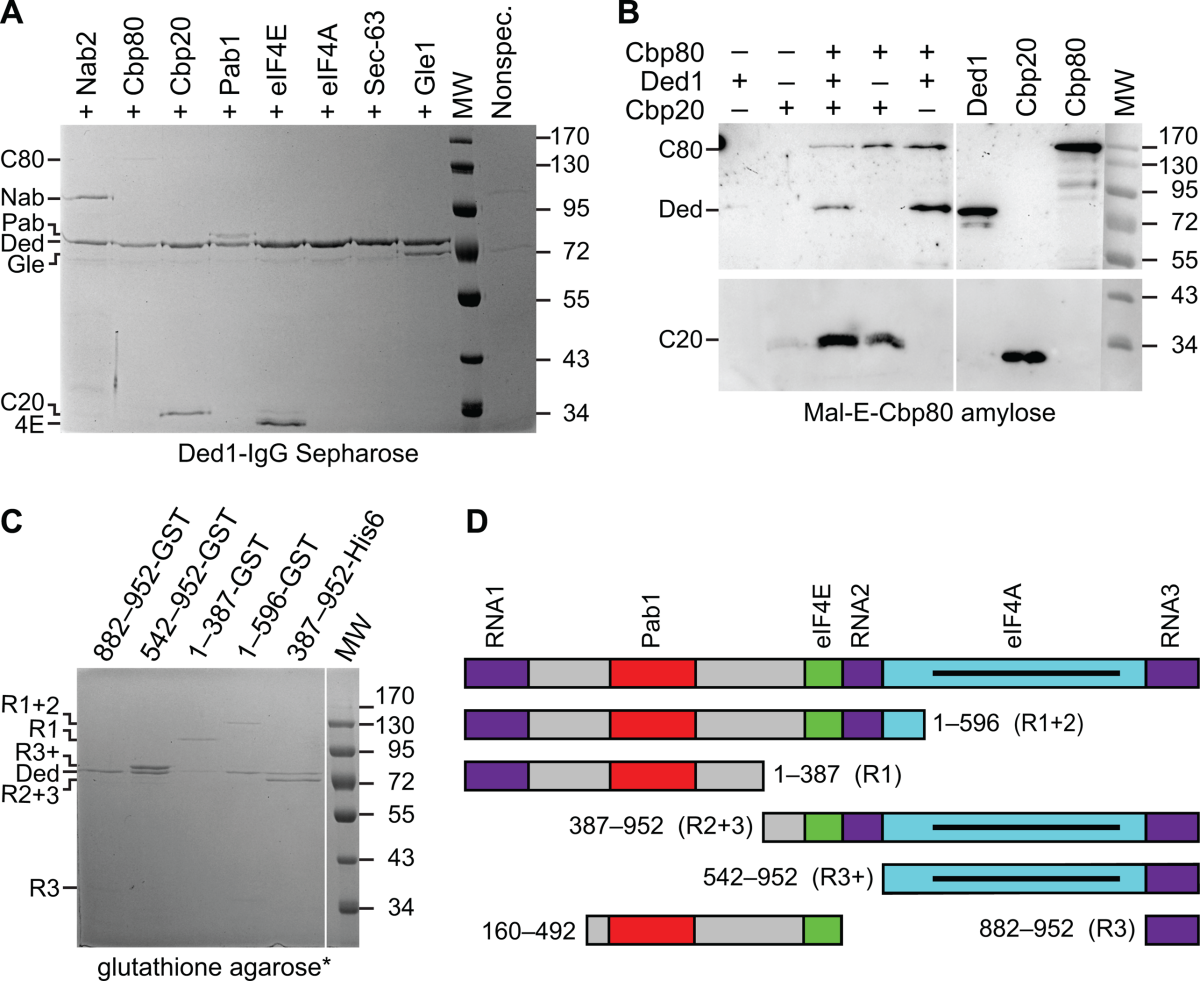
Ded1 physically interacted with the cap-associated factors. (**A**) Ded1 was bound to protein A Sepharose beads crosslinked to IgG against Ded1, incubated with the indicated purified proteins, washed and the eluted proteins separated by 10% SDS PAGE. The gel was subsequently stained with Coomassie blue. Note that Cbp80 was weakly stained, but it was clearly visible on the original gel. The ‘nonspec’ lane was a mix of all the purified proteins incubated with the resin in the absence of Ded1. (**B**) MalE-Cbp80 was bound to amylose resin beads (Biolabs), incubated with the indicated purified proteins, washed and the eluted proteins separated by 10% SDS PAGE. The gel was analyzed by western blot with IgG against Ded1, Cbp80 and the His_6_ tag of Cbp20 (Roche). Two identical gels were used to probe for Ded1 and Cbp20 because different secondary antibodies were used. Aliquots of the purified proteins (0.1 μg) are shown as markers. Note the Cbp20 protein migrated slower in the binding assay lanes due to the higher salt concentrations. (**C**) Purified GST-eIF4G1 fusion fragments bound to glutathione agarose beads, were incubated with Ded1, washed, eluted with reduced glutathione and separated by 10% SDS PAGE, which was subsequently stained with Coomassie blue. *The exception was the eIF4G-His_6_ fragment 387–952 that was retained by Ded1 bound to IgG-protein A Sepharose beads as in panel (A). Note that the 882–952 fragment gave a weak signal that was clearly visible on the original gel. For unknown reasons, the 1–387-GST fragment migrated as an aberrantly large protein. (**D**) The different fragments of eIF4G1 that were used in these experiments that show the locations of the previously identified binding motifs. RNA1–3 motifs bind the mRNA; motifs Pab1, eIF4E and eIF4A form direct protein–protein interactions with the corresponding proteins; and the solid line in the eIF4A-binding motif is the MIF4G/HEAT repeat domain that forms a protein–protein interaction surface. The markers reflect the sizes of the Prestain Protein Ladder (Euromedex).

The preceding experiments demonstrated direct physical interactions between Ded1 and cap-binding proteins. However, it was possible that some factors were indirectly associated with Ded1 through their interactions with the other proteins. Therefore, we individually tested various purified proteins for their ability to be retained by Ded1 fixed to Ded1-IgG Sepharose beads (Figure 5A). As a control, we incubated a mixture of the proteins with the beads in the absence of Ded1. Only Gle1 and Nab2 showed a weak nonspecific binding. The beads retained all of the indicated cap-associated factors in the presence of Ded1 except eIF4A. Both Pab1 and Cbp80 showed faint signals, but their presence was verified by western blot analysis. Ded1 bound to the beads also retained Gle1. This was consistent with previous observations that Gle1 physically and genetically interacts with Ded1 (75). As a control, we used the sec63 domain of Brr2 that is associated with protein–protein interactions (80); no interactions were detected, which indicated that only specific proteins were retained by Ded1. To verify the interactions between Ded1 and Cbp80, we bound a fusion construct between Mal-E and Cbp80 on amylose beads and tested for the ability to retain Ded1. Both Ded1 and Cbp20 were detected by western blot analysis in fractions containing MalE-Cbp80, but not in those lacking the fusion protein (Figure 5B). The Mal-E protein bound alone to the beads was unable to retain the proteins, which demonstrated that the interactions were mediated through Cbp80 (data not shown).

Previous workers have demonstrated a direct interaction between Ded1 and the carboxyl-terminal domain of eIF4G1 containing the RNA-binding motif RNA3 [Figure 5D; (66)]. We were interested in knowing what other regions of eIF4G1 might interact with Ded1. Thus, we constructed or obtained various constructs that expressed different fragments of eIF4G1 containing the previously identified motifs [Figure 5D; see (48) and references therein]. All the fragments bound to glutathione beads that contained the RNA-binding motifs were able to retain Ded1 with varying efficiencies (Figure 5C). These data were verified by western blot analysis for Ded1. As a positive control, we verified that the eIF4G fragments containing the eIF4E-binding motif (amino acids 1–596) and the eIF4A site (542–952) retained eIF4E and eIF4A, respectively (data not shown). In addition, Ded1 bound to IgG-protein A Sepharose beads was able to retain the carboxyl-terminal eIF4G1 fragment 387–952 (Figure 5C). The results indicated that the carboxyl-terminal region of eIF4G1 had a higher affinity for Ded1 than the amino-terminal half. In contrast, Ded1 had little or no affinity for the eIF4G9 fragment (160–492) that contained only the Pab1 and eIF4E-binding sites (data not shown). Thus, in accordance with the previously published results, we found that fragments containing the RNA3 motif formed the strongest interactions, but that there were other regions of eIF4G1 that interacted with Ded1 as well.

The same results were obtained when the pull-down experiments were done in the presence or absence of RNase I, which indicated that the interactions were probably not mediated by contaminating RNAs. Likewise, the interactions were not affected by the presence of AMP-PNP, which is a nonhydrolyzable analog of ATP that greatly enhances the affinity of Ded1 for RNAs (71). Thus, the interactions were mediated through direct protein–protein contacts, and they were not dependent on the enzymatic activity of Ded1 *per se*.

### Cap-associated factors enhanced the RNA-dependent ATPase activity of Ded1

The preceding experiments demonstrated that Ded1 formed interactions with cap-associated factors, but not with control proteins. Nevertheless, these results did not demonstrate a functional association between the different proteins. Therefore, we measured the RNA-dependent ATPase activity of Ded1 in the presence of the different purified proteins. To facilitate the analyses, we measured the reaction rates with saturating concentrations of whole yeast RNA and 1 mM ATP. The results are shown in Table 1, where 25-fold molar excesses of the indicated proteins were added to the Ded1 reactions. All of the cap-associated factors enhanced the ATPase activity of Ded1 except for eIF4E and Cbp20. Moreover, eIF4A had no significant effect (data not shown); eIF4A is a DEAD-box protein with an RNA-dependent ATPase activity, but it had <1% of the activity of Ded1 under these conditions (4). None of the other tested proteins showed significant ATPase activities in the absence of Ded1. This indicated that the preparations were free of contaminating NTPases.

**Table 1.** *In vitro* ATPase activity of Ded1

Proteins^a^	ATPase^b^	% Ded1
Ded1	76.2 ± 4.9	100
+ eIF4E	79.5 ± 4.1	105
+ eIF4G (1–596)	157. ± 5.	206
+ eIF4G (542–952)	107. ± 2.	141
+ Pab1	90.4 ± 2.	119
+ eIF4E + eIF4G (1–596)	159. ± 6.	209
+ eIF4E + eIF4G (542–952)	121. ± 5.	160
+ eIF4E + eIF4G (1–596; 542–952)	172. ± 1.	227
+ eIF4F + Pab1	192. ± 5	252
+ Cpb20	74.2 ± 1.6	97
+ Cbp80 (1–556)	168. ± 6.	220
+ Cbp80 (557–861)	110. ± 5.	144
+ Nab2	115. ± 6.	150
+ Cbp20 + Cbp80 (1–556)	180. ± 10.	237
+ Cbp20 + Cbp80 (557–861)	118. ± 6.	155
+ Gle1	145. ± 11.	191

^a^Ded1 was tested alone or with a 25-fold molar excess of the indicated proteins. The eIF4F complex consists of eIF4A, eIF4E, eIF4G (1–596 + 542–952).

^b^Reaction velocity of the RNA-dependent ATPase activity of Ded1 using 1 mM ATP shown in nM free phosphate produced per minute per nM of Ded1. Whole yeast RNA was used at 0.45 μg/μl.

We obtained good activation of Ded1 with Gle1 that was independent of added phytic acid. Gle1 in the presence of phytic acid is known to activate the intrinsically poor ATPase activity of the DEAD-box protein Dbp5 that is involved in mRNA export from the nucleus [reviewed by (6)]. However, and in contrast to our data, previously published results indicated that Gle1 actually inhibited the ATPase activity of Ded1, and that it functioned as a negative regulator of Ded1 activity (75). We have no explanation for these contradictory results. Regardless, the interactions between Gle1 and Ded1 were not the same as those with Dbp5 because the latter interactions are mediated directly through phytic acid (81).

The results were consistent with Ded1 physically and functionally interacting with the cap-associated factors, but with some unexpected effects. The cap-binding proteins eIF4E and Cbp20 had no effect on the activity, even though they physically interacted with Ded1 *in vitro*. The amino terminus of eIF4G1 (1–596) activated Ded1 better than the carboxyl terminus (542–952), even though the latter had a higher binding affinity. Of the individual proteins, the amino-terminal sequences of eIF4G1 and Cbp80 (1–556) both gave the best—and similar—stimulation, which indicated that these regions were functionally the most important. Both Pab1 and Nab2 had weak, but significant, stimulatory effects.

The stimulation of the ATPase activity was cumulative, but not additive, when different combinations of the proteins were used. The eIF4F complex of proteins with Pab1 and the CBC complex (Cbp20 + Cbp80) activated Ded1 the most. This implied that Ded1 assembled on the 7-methylguanosine of RNAs with these factors. We tested this by using a ∼200 nucleotide-long RNA (Cyc1) that we transcribed *in vitro*, polyadenylated and capped (7G-Cyc1-A). We estimated that on average ∼120–150 adenosines were added. The ATPase activation of both the unmodified Cyc1 and the modified 7G-Cyc1-A RNAs were then tested at equal molar concentrations with various combinations of the purified proteins. The Cyc1 RNA at 320 nM (0.020 μg/μl) stimulated the ATPase activity of Ded1 50% more than saturating concentrations of whole yeast RNA (0.45 μg/μl), which indicated that the latter substrate contained inhibitors as well as unproductive RNAs. The 7G-Cyc1-A RNA typically gave slightly better activation of Ded1 than Cyc1, but we attributed this to its larger size (320 nM was equivalent to 0.031 μg/μl). Regardless, no significant differences in activation were observed when the various cofactors were added. Thus, the activation of Ded1 by the cofactors was through protein–protein interactions rather than by facilitating the assembly of Ded1 on the RNA substrates. This was consistent with the observations of others that eIF4G1 forms contacts with Ded1 that are independent of the RNA (66).

The activation was highly dependent on the quantity of added protein; stoichiometric quantities showed weak activation that generally increased linearly with added protein up to the 75-fold excess that was tested. The large molar excesses needed to obtain moderate activation of Ded1 indicated that the cofactors were probably not functioning as enzymatic activators *per se*, as occurs with Dbp5 and Gle1. Instead, it appeared that the cofactors were functioning like molecular chaperones to stabilize or facilitate conformations of Ded1 that were more active. Indeed, less active preparations of Ded1 showed proportionally even larger effects for the added proteins, but they did not increase the reaction velocities beyond those measured with the more active Ded1 preparations. Thus, the cofactors may associate with Ded1 and function as allosteric activators. We concluded that the physical associations between Ded1 and the cap-associated factors were biologically significant.

### Ded1 was primarily associated with stable mRNPs not actively undergoing translation

**Figure 6.**
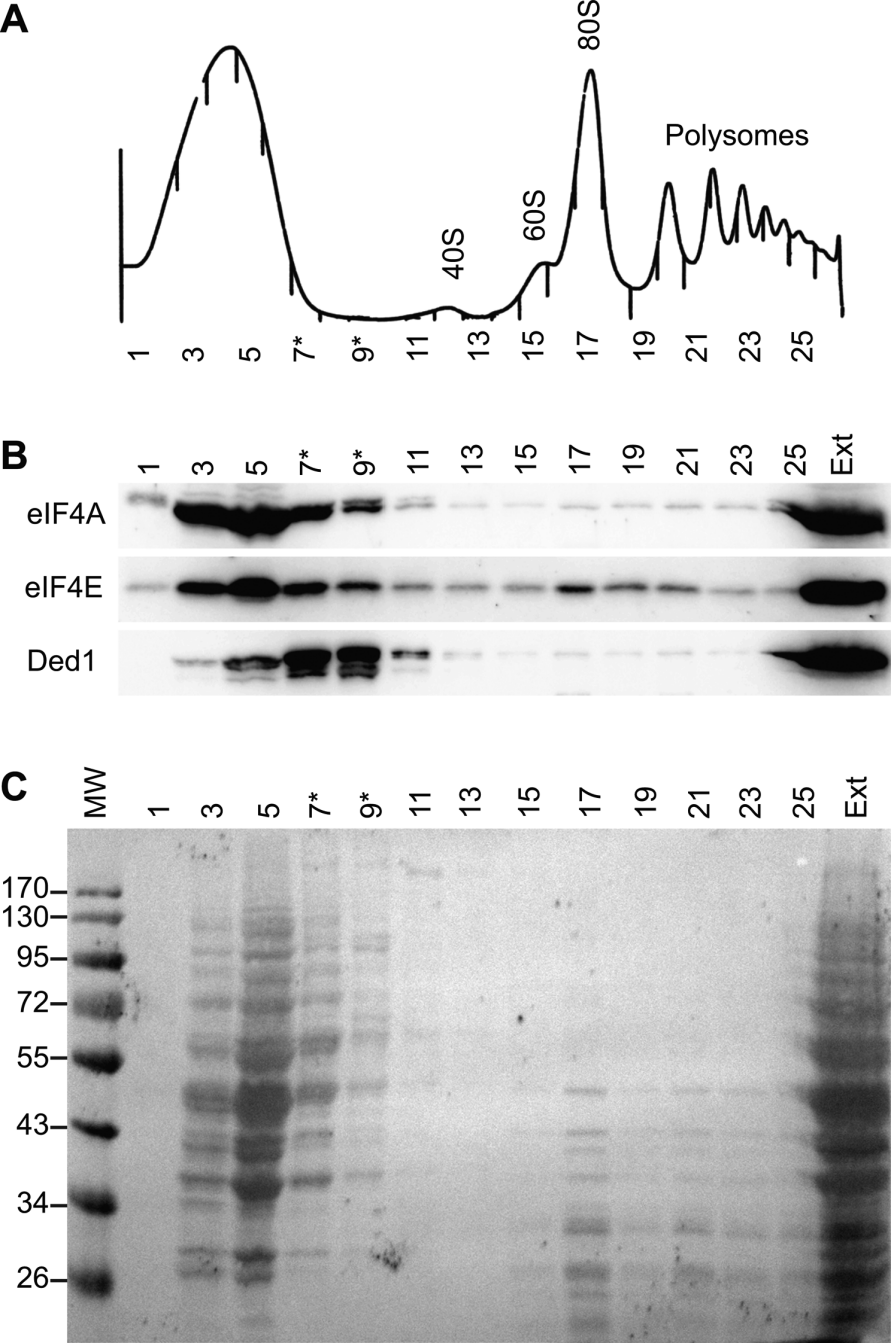
Sucrose gradients of cell extracts. (**A**) Yeast extract of the BY4742 strain was separated on a 7–47% sucrose gradient as described in the text, and ∼0.5-ml fractions were collected as indicated. The absorption profile at 254 nm is shown. Cells were incubated with 0.1 μg/ml of cycloheximide for 10 min prior to harvesting. Most of the Ded1 sedimented in complexes that were larger than those for the majority of the other detected proteins (indicated as *). (**B**) Western blot analysis of the fractions separated on a 10% SDS PAGE. Every other fraction was loaded so that the entire profile could be shown on a single gel. Ext, 10% of the original extract was loaded on the gel as a reference. (**C**) Ponceau red stained membrane of the electrophoretically transferred proteins from the SDS PAGE. MW, molecular weight markers shown in kDa.

Ded1 has been proposed to associate with the 43S ribosome late during its assembly on the eIF4F translation-initiation complex, but prior to ribosome scanning to the AUG start codon, and to promote subsequent formation of the 48S ribosome [see (76) and references therein]. Ded1 is thought to enhance scanning of mRNAs and particularly those with long, structured, 5′ UTRs. Our results suggested that Ded1 associated with cap complexes on mRNAs very early and perhaps even before transcription was terminated. To help clarify this apparent contradiction, we undertook polysome analyses of yeast cells growing in early exponential phase (OD_595_ ∼0.8). Extracts were loaded on sucrose gradients and the complexes separated by centrifugation. Fractions were collected across the gradients, the recovered proteins separated by SDS polyacrylamide electrophoresis and then the gel was subjected to western blot analysis with IgGs against various proteins (Figure 6; Supplemental Figure S6A).

We detected all the proteins throughout the gradients with the exception of Cbp20, which showed no detectable signal in the 80S or polysome region. However, the vast majority of the signals were found close to the top of the gradients, which indicated that they were not actively engaged in translation. Based on the amount of Ded1 in the loading extract, we recover the vast majority of Ded1 in these fractions (>80–90%). The peak intensities for most of the proteins coincided. The peak for eIF4G1 also corresponded to the other proteins, but the western blot showed multiple fragments, which indicated that it was degraded during the analyses. Ded1 is thought to associate with another DEAD-box protein, Dhh1, in cells under conditions of stress or after post-diauxic shift (22,74,82). Dhh1 is involved in translational repression and mRNA decay (83). We found that under our conditions that Dhh1 sedimented at the same size range as for the other factors. In contrast, the peak for Ded1 was reproducibly shifted toward larger complexes in a region of the gradient that contained relatively little protein (Figure 6, Supplemental Figure S6). Thus, Ded1 appeared to be associated with a subset of the RNPs.

It is known that the pre-initiation complexes are not completely resistant to the conditions used to extract the cells and run the gradients (84). Therefore, it was possible that the proteins profiles we obtained were a result of the partial dissociation of larger complexes. We tested this for Ded1 by analyzing polysome profiles of cells first treated, *in vivo*, with formaldehyde to stabilize the complexes. Under these conditions, we did see more Ded1 associated with the polysomes and a slight peak around the 40S ribosome, but the majority of the Ded1 migrated as before (data not shown). Thus, we concluded that the observed complexes with Ded1 were only partially the result of the dissociation of larger complexes. In contrast, the majority of the other factors sedimented in a region of the gradient that contained a large amount of proteins (Figure 6). Only eIF4E showed a slight peak in the 80S region of the gradient, which was consistent with its role as a cap-binding protein. The translation-initiation factor eIF4A is thought to dissociate from the mRNA prior to 80S assembly (76), and it is not associated with the polysomes. Likewise, the CBC and Dhh1 proteins were not expected to cosediment with translating ribosome. Thus, and in contrast to Ded1, we could not demonstrate that a majority of these factors were associated with stable RNPs.

It was possible that Ded1 transiently associated with mRNAs during the early phases of translation initiation that preceded 40S assembly on the mRNA, as previously proposed (66). Therefore, we repeated the polysome analysis but with cells first treated with rapamycin. Rapamycin blocks the TOR1-dependent signaling pathways, which subsequently inhibits a number of cellular processes that include translation initiation [reviewed by (85)]. We reasoned that if Ded1 interacted with the cap-associated factors during translation initiation, then rapamycin treatment would alter the resulting complexes. Consistent with this, rapamycin reduced the polysome profile and increased the amount of free 40S, 60S and 80S ribosomes (Supplemental Figure S6B). The western blots showed slightly altered profiles for the protein distributions, but they were consistent with the factors being in complexes that were not associated with active translation. Other workers have obtained similar results (74). However, it should be noted that the TOR2 pathway, which is involved in the expression of a subset of genes, is not affected by rapamycin treatment.

To further characterize the complexes associated with Ded1, we extracted the complexes of selected fractions with Ded1-IgG Sepharose beads and then analyzed the recovered proteins by nanoLC-MS/MS (Supplemental Figure S7). The analyses were complicated by the low recovery of some of the fractions, but we were able to identify 61 proteins in fraction 4, 82 proteins in fraction 6 and 46 proteins in fraction 7. Many of the identified proteins were unrelated to the proposed functions of Ded1, which indicated that the purification strategy was not sufficiently stringent. Nevertheless, 25% of the proteins in fraction 4, 52% in fraction 6 and 59% in fraction 7 were associated with either transcription, translation or with ribosomes. This was consistent with Ded1 being in both nuclear and cytoplasmic mRNPs. The Mascot score for Ded1 in fractions 4, 6 and 7 were 529.0, 413.4 and 368.2, respectively. We recovered eIF4E in fractions 4 and 6 with Mascots scores of 52.2 and 67.1, respectively. Pab1 was recovered in all three fractions (85.6, 385.3, 151.1). None of the other proteins identified by western blot were identified by mass spectrometry. Hence, the complete complexes associated with Ded1 may not have been stable enough to resist our purification protocol or the mass spectrometry analyses did not detect their presence. For the latter possibility, it is known that some proteins are recalcitrant to detection by mass spectrometry (86). Interestingly, we also identified four proteins of the 26S proteasome in fraction 7, which suggested that fraction 7 contained complexes that sedimented at a size corresponding to 26S (Supplemental Figure S7). Finally, we verified the presence of polyadenylated RNAs by northern blot analyses of the extracted fractions with a ^32^P-labeled (dT)_25_ probe (data not shown). These data were consistent with Ded1 associating with different mRNP complexes in the nucleus and cytoplasm, and they were consistent with Ded1 being associated with the remodeling events of these mRNPs.

## DISCUSSION

### Ded1 is a cap-associated factor that shuttles between the cytoplasm and nucleus

**Figure 7.**
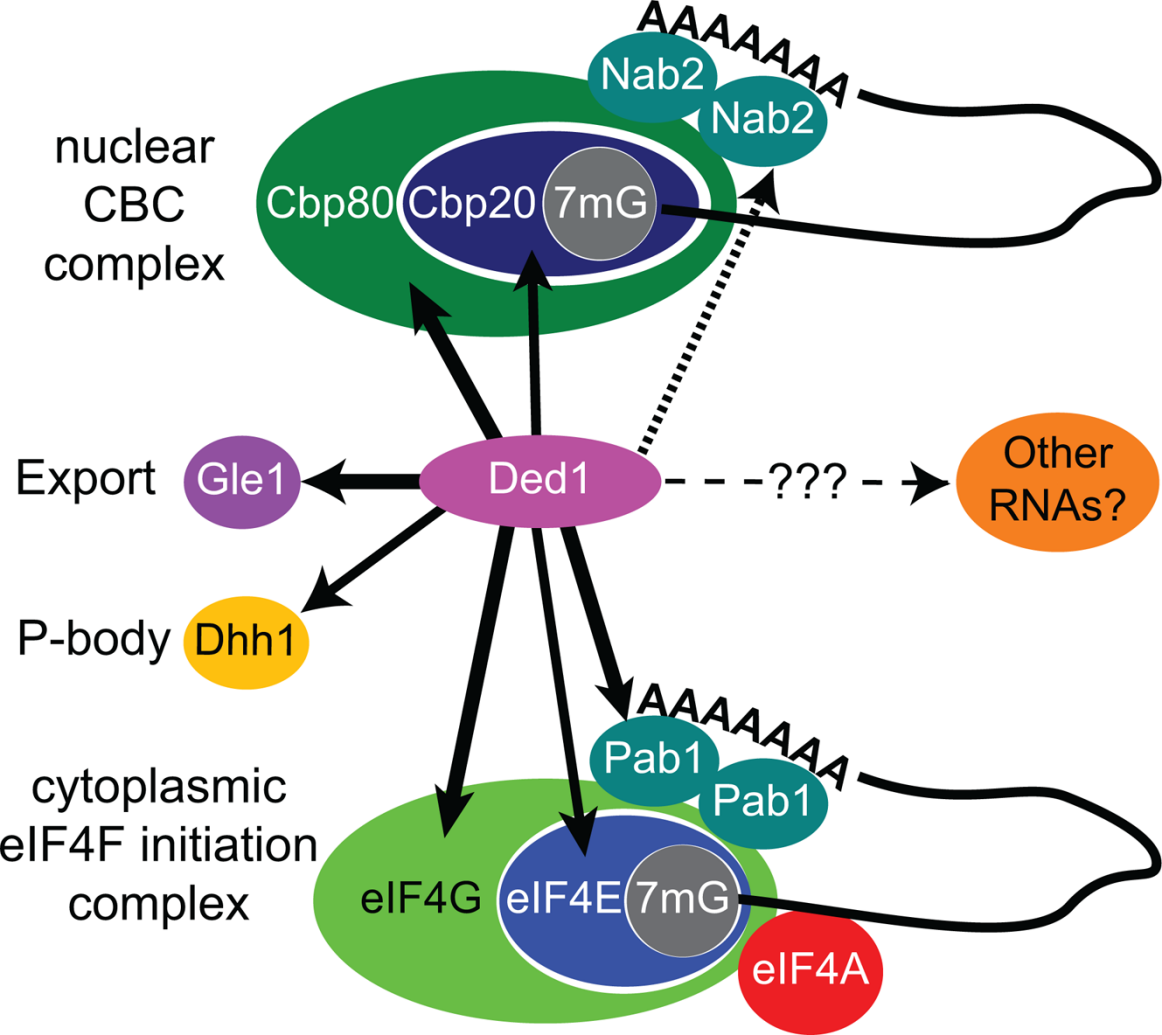
Protein partners of Ded1. Ded1 interacts with the predominately nuclear CBC complex factors, which consists of Cbp20 and Cbp80, and with Nab2. Ded1 also interacts with the predominately cytoplasmic translation-initiation factors, which includes Pab1. We have no evidence that Ded1 interacts with the DEAD-box protein eIF4A, which is part of the translation-initiation complex eIF4F. Moreover, Ded1 interacts with the DEAD-box protein Dhh1, which is often localized in P-bodies in the cytoplasm. It is involved in mRNA decapping and degradation. Gle1 is located on the cytoplasmic side of the nuclear pore, and it is involved in the nuclear export of mRNAs. However, Gle1 also has been implicated in regulating translation. The heavy lines represent interactions determined by physical, genetic and enzymatic approaches, the medium line by physical and genetic approaches, and the medium dotted line by physical and enzymatic approaches. The light dotted line indicates other potential substrates. Interactions were determined by this work and the published work of others (see text).

Our experiments demonstrate that Ded1 is a physical and functional component of the nuclear and cytoplasmic cap-binding complexes. We find that Ded1 is genetically linked to various CBC and eIF4F proteins, and that it physically interacts with these proteins *in vitro* (Figure 7). Moreover, we find that these proteins enhance the RNA-dependent ATPase activity of Ded1 *in vitro*. The Ded1-GFP protein actively shuttles between the nucleus and cytoplasm using both the Crm1- and Mex67-dependent nuclear export pathways, which is largely independent of the enzymatic activity of Ded1. Thus, Ded1 is probably a component of RNPs located both in the nucleus and the cytoplasm. Our mass spectrometry analyses indicate that Ded1 is associated with a large number of other proteins in cell extracts and thus in multiple different RNPs. Finally, sucrose gradients reveal that Ded1 cosediments with known cap-associated factors but that the peak concentrations of the factors do not coincide, which indicates that Ded1 interacts with a subset of these factors that form larger complexes. This distribution is not profoundly affected with rapamycin treatment, which indicates that the majority of the RNPs associated with Ded1 are not actively being translated.

These results are consistent with previous observations that Ded1 is linked with processing events that extend from transcription, splicing and translation to RNA degradation (16–19,22). They also are consistent with crosslinking experiments that demonstrate a physical interaction between Ded1 and RNA polymerase II *in vivo* (87). And they are consistent with microarray experiments, which compared mRNA and pre-mRNAs levels, which implicate Ded1 as a spliceosome factor that influences splicing (88). Similar results were obtained for DDX3X (68,69). Although we limited our studies to cells growing under optimal conditions (exponential growth), other workers have demonstrated that Ded1 is found in specialized cytoplasmic granules of aggregated, translationally-inactive, mRNPs, known as P-bodies and stress granules, under conditions of stress (22,66,74,82).

### Ded1 is associated with mRNPs that undergo multiple remodeling events

Cap-associated complexes are important mediators in the control of gene expression [reviewed by (9,67)]. The 7-methyl guanosine cap is added to the 5′ ends of pre-mRNAs cotranscriptionally when the nascent RNAs are 22–25 nucleotides long. The cap-binding proteins Cbp20 and Cbp80 associate with the RNAs shortly thereafter and remain associated with the RNAs during the different processing events that include splicing, polyadenylation, quality control and RNA export. In mammalian cells, the CBC proteins are still associated with the mRNAs during the pioneering round of translation in the cytoplasm as part of the quality control processing of the mRNAs [reviewed by (89)]. Yeast differs from the mammalian systems in some respects (e.g. there is no EJC), but the exported, CBC bound, mRNPs also are thought to undergo a pioneering round of translation (90). Subsequently, the various nuclear factors are replaced by the cytoplasmic equivalents, and the eIF4F-associated mRNAs undergo steady-state rounds of translation. At the end of their life spans, the mRNAs are deadenylated, the eIF4F complex dissociated, the cap removed and the resulting RNA degraded by exonucleases, which often occurs in the P-bodies [reviewed by (91)].

Each of the described processing events involves significant remodeling of the mRNPs. Our results, and the results of others, indicate that Ded1 is associated with many, if not all, of these different mRNP complexes. However, these experiments do not distinguish between active or passive role(s) for Ded1 nor do they reveal whether Ded1 remains fixed to each mRNP throughout its life span. It is possible that Ded1 dissociates and then reassociates with the mRNPs during the different remodeling events in the different cellular compartments. This latter possibility is consistent with the proposed role of Ded1 as a regulator of translation initiation (20,22,66,75). Nevertheless, we have not yet been able to demonstrate a direct enzymatic role for Ded1 in the formation of these RNPs.

### Ded1 homologs in other organisms regulate gene expression

We have shown that mouse PL10 and human DDX3X are able to complement a deletion of Ded1 in yeast fairly well while Belle and Vasa show more limited effects. Others have shown that *S. pombe* Ded1 also will support growth in *S. cerevisiae* (52). The *S. pombe* Ded1 is implicated in cell cycle regulation (52,92). It was shown to interact with the protein kinase Chk1, which is involved in checkpoint response, and with the cyclin-dependent kinase Cdc2, which is involved in regulating the cell cycle (93). DDX3X has multiple functions in gene regulation, cell cycle control and viral replication [reviewed by (26,94)]. More recently, DDX3X was shown to regulate cell growth by controlling translation of cyclin E1 (95) and as a regulatory subunit of casein kinase 1 in Wnt-β-catenin signaling (96). Interestingly, the latter function was independent of the enzymatic activity of DDX3X. DDX3X also was found to bind eIF4E and act as a negative inhibitor of translation by preventing eIF4E from interacting with eIF4G in a manner analogous to the antagonistic roles of the 4E-BP proteins (97). Moreover, DDX3X enhanced the affinity of eIF4E for the cap. This interaction is likewise independent of the enzymatic activity of DDX3X. These results are contrary to those previously determined for Ded1 because Ded1 stimulates eIF4E-dependent translation; eIF4E is inhibited by p20, which is the yeast equivalent of the 4E-BP proteins (19). By these criteria, Ded1 would be a positive regulator of translation, while DDX3X is a negative regulator. However, other experiments show a stimulatory role of DDX3X in translation while Ded1 can be inhibitory (65,66). Moreover, DDX3X can substitute for eIF4E under certain conditions, although we have no evidence that this is true for Ded1 as well (98).

As seen for Ded1, DDX3X is found in stress granules under conditions of stress. DDX3X is thought to promote stress granule formation through its interactions with eIF4E and Pabp1 (99). Others have used confocal microscopy to show that DDX3X is associated with various translation-initiation factors in stress granules (65). DDX3X is ineffectual at promoting scanning of 5′ UTRs in *in vitro* reconstitution experiments in contrast to Ded1, which enhances the scanning of mRNAs with moderately stable basepaired stems (100). Finally, DDX3X is thought to promote the translation of specific mRNAs with structured 5′ UTRs, where it binds the mRNA and clamps the entry of eIF4F through its interactions with eIF4G and Pabp1 (65). These interactions are only needed in the very early steps of ribosome binding and prior to 43S ribosomal scanning. This latter result points to a role for DDX3X in RNP assembly rather than as a processive helicase to disrupt RNA secondary structures that would impede ribosomal scanning. On the whole, these data are consistent with the Ded1 subfamily of proteins being needed to positively or negatively regulate the expression of specific genes that are used to control the cellular response to growth conditions and the cell cycle.

### What is the role of Ded1 in yeast?

Ded1 was originally considered as a general translation-initiation factor that was needed to promote 43S ribosome scanning by disrupting RNA secondary structures in the 5′ UTRs (18,19). Later work indicated that Ded1 associated with the 43S ribosome late during translation initiation to promote scanning of mRNAs with long, structured, 5′ UTRs and formation of the 48S ribosome (20,76). Ded1 has been considered a bifunctional protein that enhances translation initiation but at the same time can repress translation by promoting the sequestering of mRNPs in P-bodies—a process that, like for DDX3X, is independent of the ATPase activity of Ded1 (22,82). Others have found that Ded1 interacts early with eIF4G to assemble the mRNP translation-initiation complex, which accumulates in stress granules (66). Thereby, Ded1 can function as a repressor of translation by forming mRNPs stalled in translation initiation and as an activator by releasing the stalled mRNPs from the stress granules in an ATP-dependent fashion. Finally, Gle1 was found, in contrast with our results, to be a negative regulator of Ded1 in translation initiation through direct protein–protein interactions (75). Nevertheless, Ded1 has been implicated in transcription and splicing as well (16,17,87,88).

Our experiments indicate that Ded1 associates with the cap complexes of a subset of mRNPs very early in the life of the mRNAs in the nucleus, and that these mRNPs are subsequently exported to the cytoplasm. However, we cannot be sure of the role that Ded1 plays in these different complexes or even if Ded1 remains associated with the same mRNA throughout the processing events. It is possible that Ded1 is needed to provide the energy necessary to drive the remodeling of the different RNPs. For example, Cbp20 binds the cap structures of mRNAs with up to 40-fold higher affinity than eIF4E (101). Thus, the remodeling of the CBC complex into the eIF4F initiation complex would require a substantial amount of energy that could be provided through the ATPase activity of Ded1. Likewise, energy would be required to drive the other remodeling events to completion in a unidirectional fashion. However, there are other NTP-dependent DExD/H-box proteins implicated in these processes as well, so we cannot attribute a specific role for Ded1. Nevertheless, our experiments, and the experiments of others, strongly support a role of Ded1 in the regulation of gene expression of a subset of proteins that are probably involved in controlling the cellular response to the growth conditions and in regulating the cell cycle. Ded1 may associate with specific mRNAs linked with these proteins and mark them for processing, export, translation, storage or degradation. Ded1 may drive the remodeling events of the mRNPs needed to express the genetic information or alternatively it may become activated by the remodeling events to promote, for example, the formation of the translation-initiation complex. Additional experimentation is needed to clarify all this, but it appears clear that Ded1 serves an important role or roles very early in the lifespan of the mRNAs.

## SUPPLEMENTARY DATA

Supplemental data are available at NAR Online.

SUPPLEMENTARY DATA
