# Supplementary material for: The DEAD-box helicase Ded1 from yeast is an mRNP cap-associated protein that shuttles between the cytoplasm and nucleus
Source: Nucleic Acids Res. 2014 Jul 10;42(15):10005–22. doi: 10.1093/nar/gku584 (PMC4150762; doi:10.1093/nar/gku584)
Supplement: SUPPLEMENTARY DATA [file supp_42_15_10005__index.html]

The DEAD-box helicase Ded1 from yeast is an mRNP cap-associated protein that shuttles between the cytoplasm and nucleus — SUPPLEMENTARY DATA 

# The DEAD-box helicase Ded1 from yeast is an mRNP cap-associated protein that shuttles between the cytoplasm and nucleus

## SUPPLEMENTARY DATA

**Files in this Data Supplement:**

- Supplementary Data
