# Supplementary material for: The DEAD-box helicase Ded1 from yeast is an mRNP cap-associated protein that shuttles between the cytoplasm and nucleus
Source: Nucleic Acids Res. 2014 Jul 10;42(15):10005–22. doi: 10.1093/nar/gku584 (PMC4150762; doi:10.1093/nar/gku584)
Supplement: SUPPLEMENTARY DATA [file supp_gku584_nar-03175-a-2013-File009.pdf]

NAR Supplemental data 2014

The DEAD-box helicase Ded1 from yeast is an mRNP cap-associated protein that shuttles between the cytoplasm and nucleus.

Meriem Senissar, Agnès Le Saux, Naïma Belgareh-Touzé, Céline Adam, Josette Banroques and N. Kyle Tanner

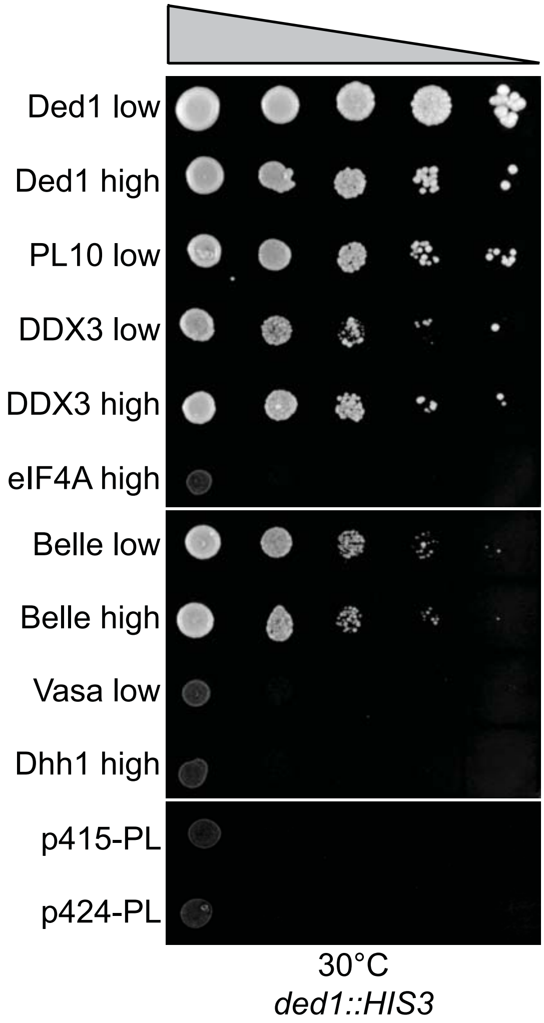

Supplemental Figure S1. Ded1 homologs complemented a yeast strain deleted for *DED1* (*ded1::HIS3*). Cells containing the *DED1*-p416 *URA3* plasmid were transformed with plasmids containing the genes encoding the indicated proteins. Overnight cultures were serially diluted by factors of ten and spotted on synthetic-medium dextrose-agar plates containing 5-FOA, which selected for cells that no longer harbored the *URA3* plasmid. Plates were incubated for 3 days at 30°C. "High" refers to expression from 2-μm multicopy plasmids while "low" was expression from low-copy centromeric plasmids; except for Ded1, both types of plasmids gave similar phenotypes. The plasmid overexpressing the unrelated DEAD-box proteins eIF4A and Dhh1, and the empty plasmids p415-PL and p424-PL were used as negative controls.

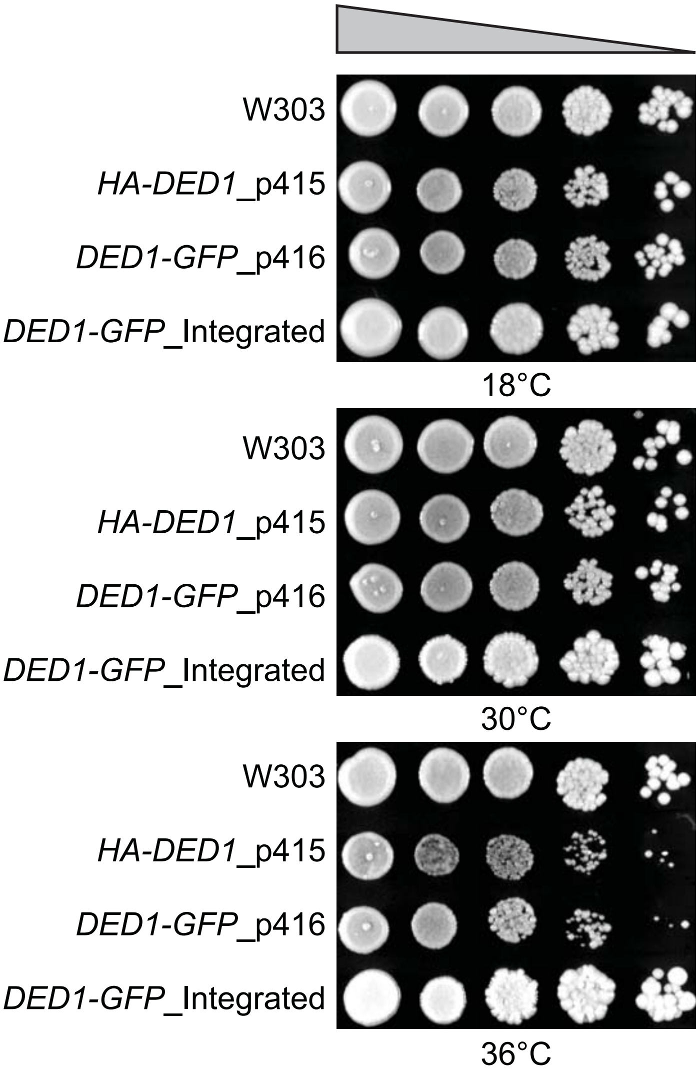

Supplemental Figure S2. The fusion *DED1-GFP* complemented a yeast strain deleted for wildtype *DED1*. Cells deleted for (d*ed1::HIS3*) in the W303 strain were transformed with the *DED1-GFP*-p416 *URA3* or *HA-DED1*_p415 *LEU2*, low-copy, centromeric plasmids containing constitutive ADH promoters, and then overnight cultures were serially diluted by factors of ten and spotted on agar plates containing yeast extract, peptone and dextrose (YPD). Plates were incubated for 3 days at 30°C and 36°C and for 7 days at 18°C. The W303 series were wildtype cells. The plasmid-encoded proteins supported slower growth at the higher temperature. In contrast, the *DED1-GFP* fusion integrated into the chromosome under the *DED1* promoter of the G50 strain, which is a derivative of W303, fully complements yeast growth at all temperatures. Thus, the slightly reduced growth at 36°C was due to the expression levels of Ded1 rather than to perturbations of the protein function caused by the GFP fusion or HA tag.

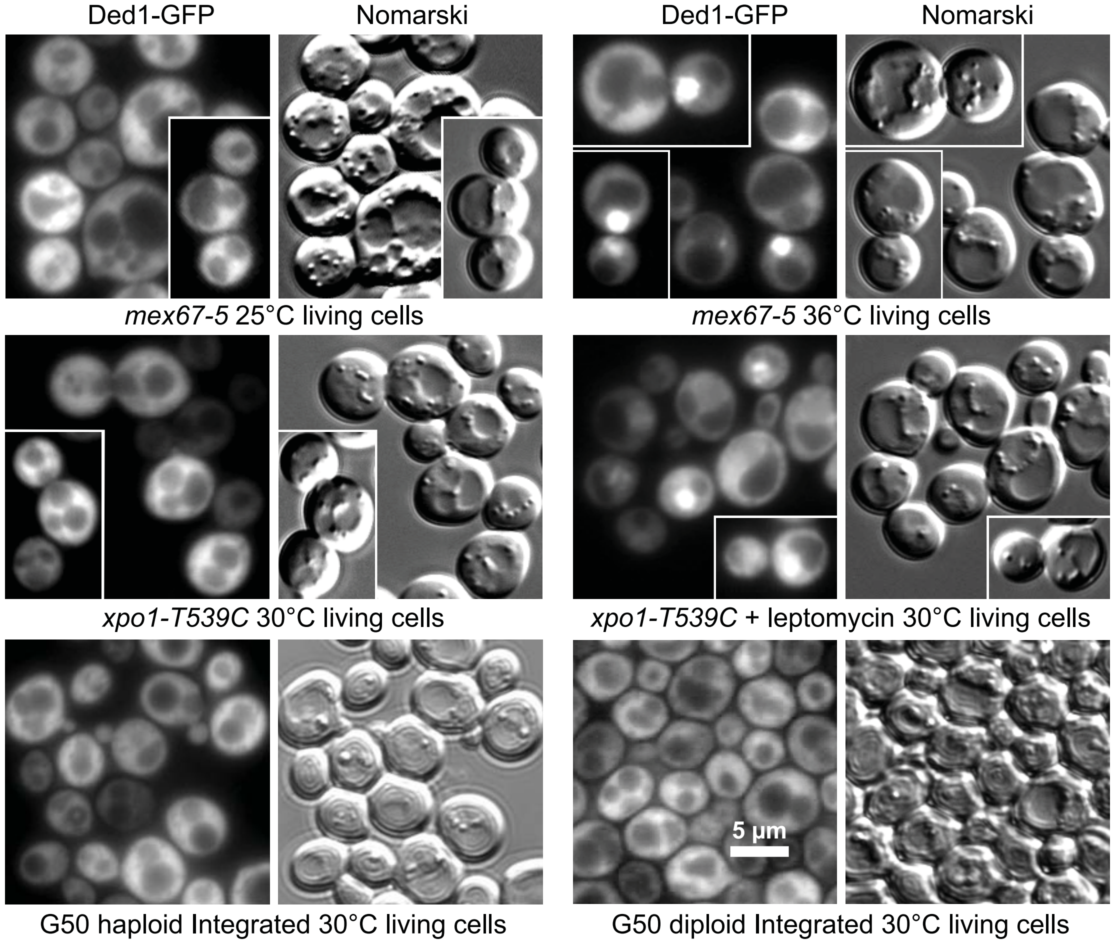

Supplemental Figure S3. Ded1shuttled between the nucleus and cytoplasm by both the Mex67 and Xpo1 nuclear-pore pathways. We obtained a yeast strain deleted for *MEX67* but that expressed a plasmid-encoded, temperature-sensitive mutant of the protein (*mex67-5*). When transformed with the *DED1-GFP* encoded on the p416 plasmid, cells showed a predominately cytoplamic location at the permissive temperature (25°C), but some cells showed a strong nuclear accumulation at the nonpermissive temperature (36°C). Similarly, a yeast strain expressing a leptomycin-sensitive form of Xpo1 (*xpo1-T539C*) showed a strong accumulation of Ded1-GFP in the nucleus of some cells in the presence of leptomycin. *DED1-GFP* integrated into the chromosome of both haploid and diploid cells showed a similar distribution of Ded1-GFP as the plasmid-encoded variants.

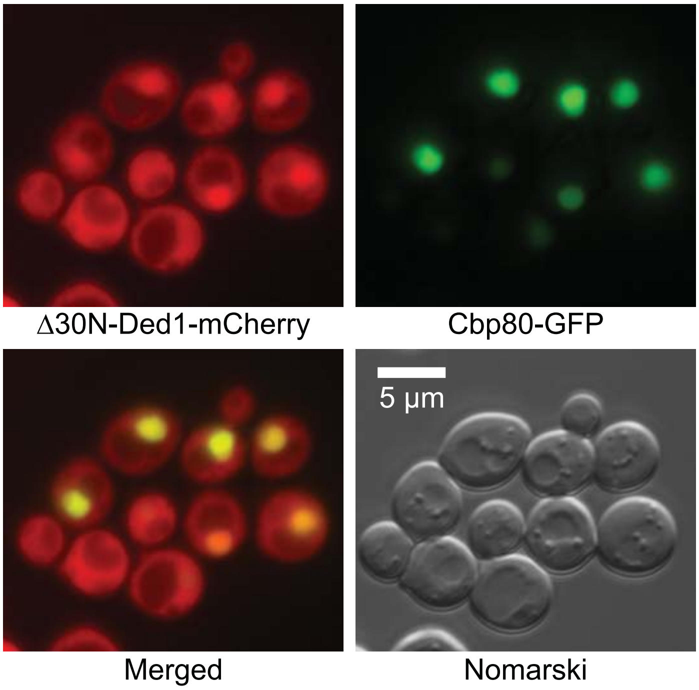

Supplemental Figure S4. Ded1 deleted for the 30 amino-terminal residues colocalizes with Cbp80 in the nucleus. Δ30N-Ded1-mCherry was coexpressed with Cbp80-GFP in the wildtype G50 strain from p416 and p414 ARS-CEN plasmids, respectively, containing ADH promoters.

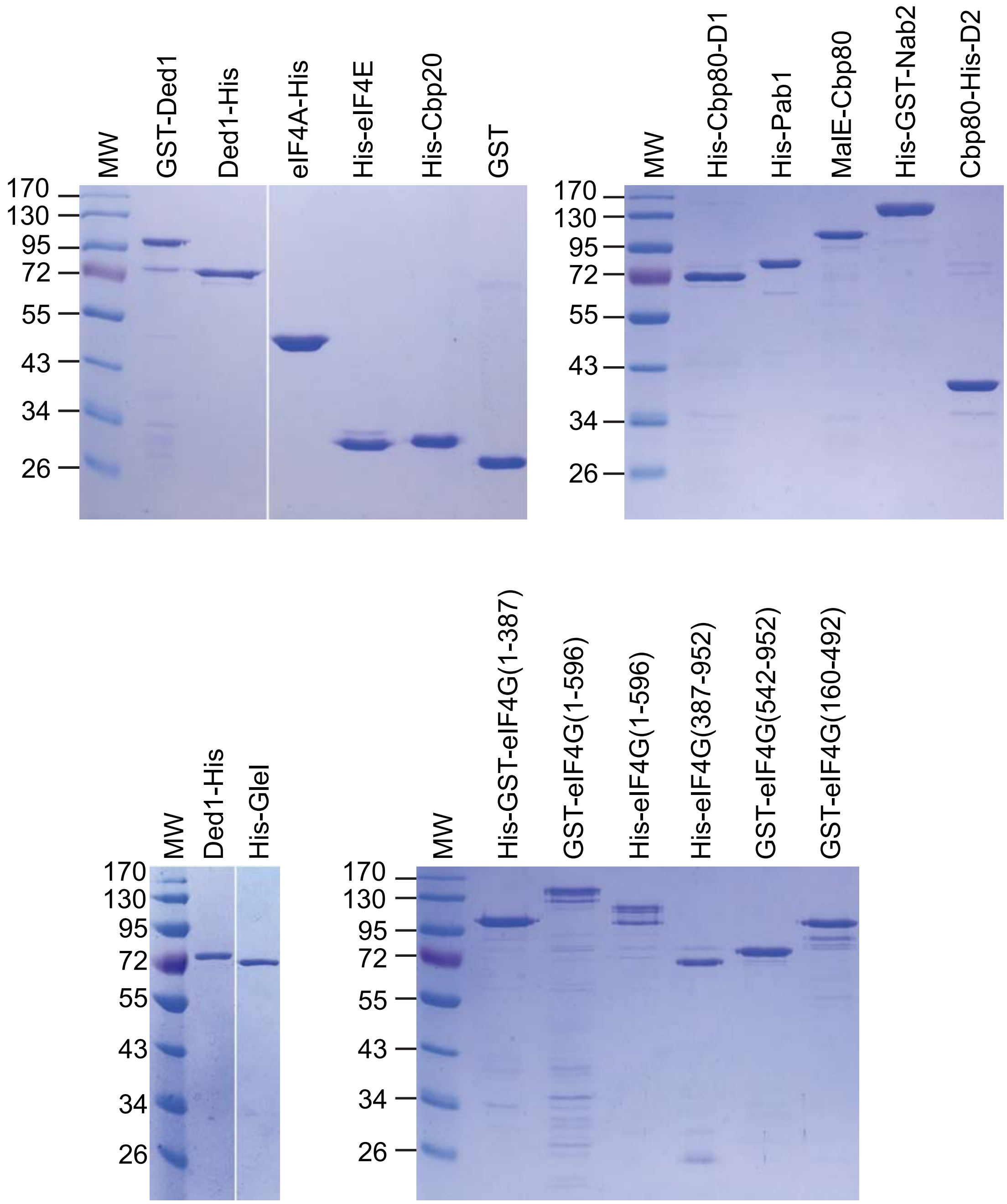

Supplemental Figure S5. Purified recombinant proteins. Recombinant proteins were expressed in *E. coli* and purified as indicated in Material and Methods in the primary text. Proteins were electrophoretically separated on a 10% polyacrylamide gel containing SDS. The gel was subsequently stained with Coomassie Blue R250. Note that the amino-terminal region of eIF4G1 contains extensive regions of intrinsic disorder, and subsequently the purified fragments showed more degradation.

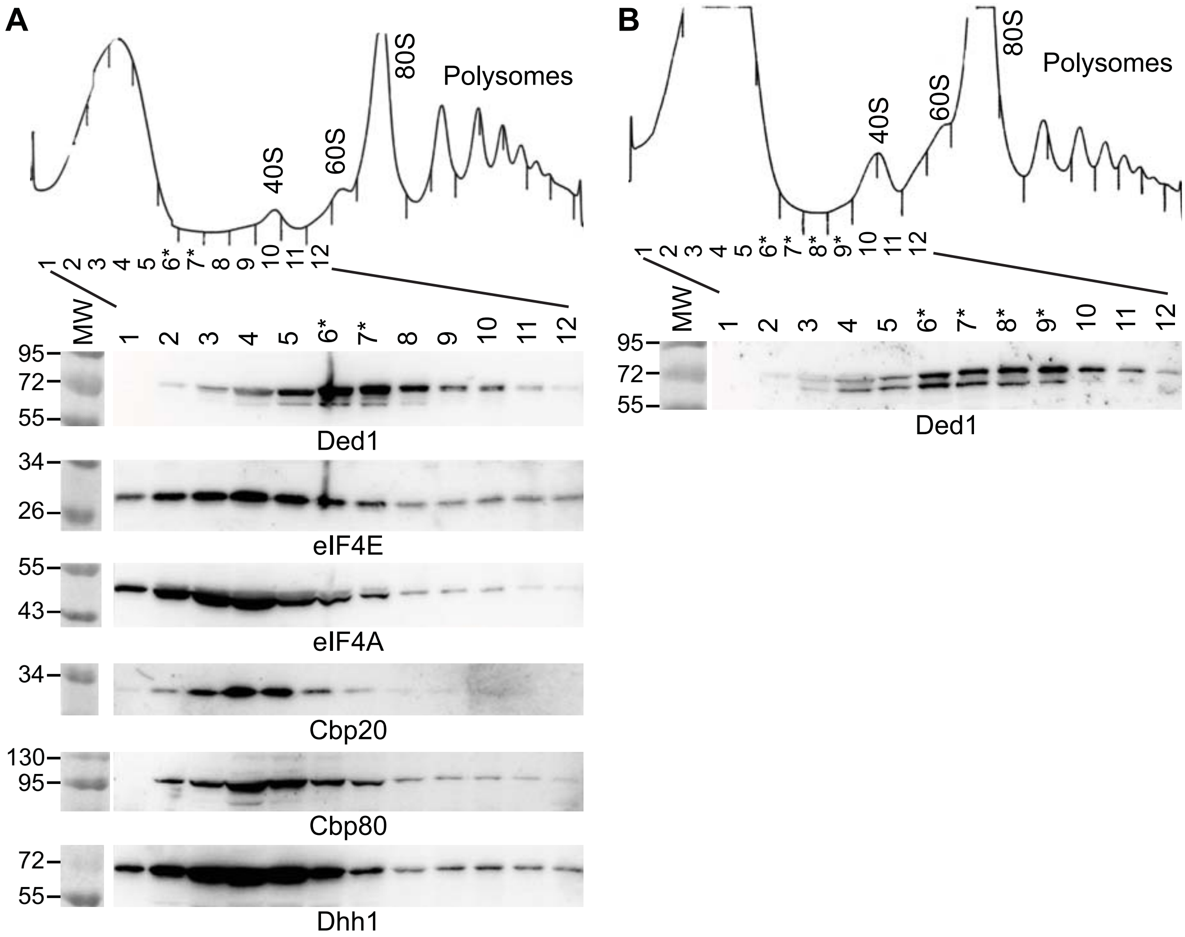

Supplemental Figure S6. Sucrose gradients of cell extracts. Cells were treated with cycloheximide to block translation elongation and then extracts were separated on 7–47% sucrose gradients. Fractions (0.5 ml) were collected and monitored spectroscopically at 254 nm. The proteins from the resulting fractions were separated on 10% SDS PAGE, transferred to membranes and probed with IgG against the indicated proteins. The majority of Ded1 (indicated by the *) sedimented in regions of the gradient with relatively little absorption, which indicated that Ded1 was in a minor subset of the RNPs. (A) Extracts from wildtype cells growing at early exponential phase. (B) Extracts from cells treated with rapamycin prior to harvesting, which blocks translation initiation in the TOR1 pathway. Thus, most of the Ded1 was in stable complexes that were not actively undergoing translation initiation. Note that we do not consider the broading of the Ded1 peak significant because the large amount of protein near the top of the gradient altered the sedimentation characteristics slightly. The markers reflect the sizes of the Prestain Protein Ladder (Euromedex).

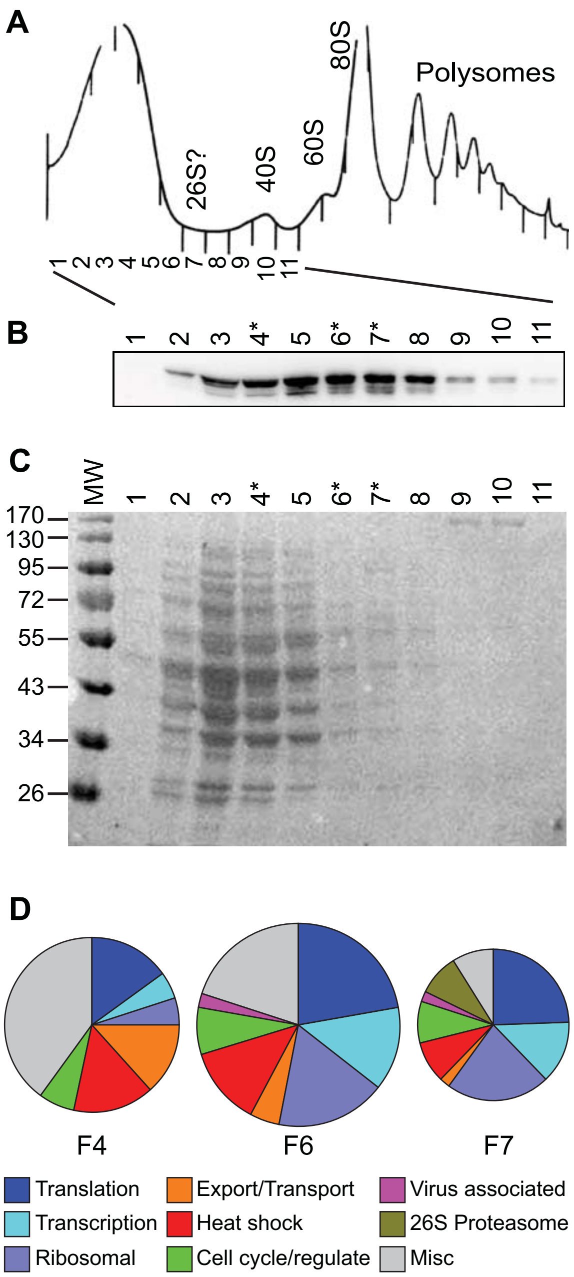

Supplemental Figure S7. Ded1 is associated with complexes involved in various processes. (A) Absorption profile at 254 nm of a sucrose gradient (7–47%) of cell extracts, where cells were incubated with cycloheximide prior to extraction. The numbers indicate the positions of the 0.5 ml fractions collected for further analysis. (B) Western blot analysis of aliquots of the fractions using IgG against Ded1. The vast majority of Ded1 (>90%) was found in these fractions. (C) Ponceau S red-stained Western blot prior to incubation with IgG that shows the protein distribution corresponding to the absorption profile. (D) Identified proteins. Fractions 4, 6 and 7 (*) were incubated with IgG against Ded1 that were crossslinked to protein A Sepharose beads, washed, eluted with glycine, precipitated as described in the text and analyzed by nanoLC-MS/MS. We identified 61 proteins in fraction 4, 82 in fraction 6 and 46 in fraction 7; the size of the plots correspond to the quantity of proteins identified. The identified proteins were classified with SlimMapper (http://www.yeastgenome.org/cgi-bin/GO/goSlimMapper.pl).

Table S1: Ded1-IgG Pull-down Nano-LC ESI MS/MS

Protein	Meta Score[a]	#Spectres[b]	SC%[c]	RMS (ppm)[d]
Ded1	6259.7	635	94.5	5.12
eIF4A	2714.1	159	65.6	2.66
Pab1	1554.5	74	45.4	3.55
Dhh1	1503.5	66	40.7	2.16
eIF4G1	1289.1	65	27.4	1.34
eIF4E	604.1	39	38.0	3.47
eIF4G2	125.3	6	4.3	1.41
Cbp20	74.2	4	20.7	2.92

[a]Mascot probably-based scoring.
[b]Spectral counting; the same peptide is fragmented up to six times over a mean elution time of 30 seconds.
[c]Percentage of the protein sequence covered.
[d]Mean error in ppm.

Table S2: Pre-Immune-IgG Pull-down Nano-LC ESI MS/MS

Protein	Meta Score[a]	#Spectre[b]	SC%[c]	RMS (ppm)[d]
Ded1	1832.5	114	46.2	2.72
eIF4A	438.0	14	20.8	4.85
eIF4E	111.3	3	10.8	2.00
Dhh1	47.4	2	2.2	4.68

[a]Mascot probably-based scoring.
[b]Spectral counting; the same peptide is fragmented up to six times over a mean elution time of 30 seconds.
[c]Percentage of the protein sequence covered.
[d]Mean error in ppm.
